# New data on the hoverflies of Morocco (Diptera, Syrphidae) with faunistic and bibliographical inventories

**DOI:** 10.3897/zookeys.971.49416

**Published:** 2020-09-29

**Authors:** Souad Sahib, Ouafaa Driauach, Boutaïna Belqat

**Affiliations:** 1 Department of Biology, Faculty of Sciences, University Abdelmalek Essaâdi, Tétouan, Morocco University Abdelmalek Essaâdi Tétouan Morocco

**Keywords:** Checklist, Diptera, hoverflies, Morocco, new records, North Africa, Palaearctic region, Syrphidae

## Abstract

All published records of 148 species of hoverflies from Morocco are reviewed and appropriate literature references, new locality records, and relevant comments are provided for each species. The list is supplemented with records from new field surveys. Two species, *Eumerus obliquus* (Fabricius, 1805) and *Orthonevra brevicornis* Loew, 1843 are recorded for the first time in Morocco. The new checklist comprises 150 nominal species from three subfamilies, 14 tribes, and 49 genera.

## Introduction

Syrphidae (Diptera: Brachycera), commonly named hoverflies, constitute, among the order Diptera, a taxonomically well-characterised family. Most adults feed on pollen and nectar and thus are potentially important pollinators and their conservation is essential to sustain the productivity of natural and agricultural landscapes (Moquet et al. 2018). The larvae of Syrphidae have various food habits; certain species are predators while others are phytophagous, mycophagous or saprophytophagous (Rotheray and Gilbert 1999, Rotheray 1993, Büchs 2003).

Hoverflies occur in all geographical regions except for Antarctica and some isolated islands. They are found from sea level to the highest altitudes in flower-rich habitats. The family contains more than 6,100 species Worldwide (Pape et al. 2011). Of the North African countries bordering the Mediterranean Sea, Morocco is the one best known faunistically. Since Becker and Stein (1913) published an initial list of 42 species, several authors have contributed to the knowledge of Moroccan Syrphidae (Gil Collado 1929, Séguy 1930, 1934, 1941, 1949, 1953, Kanervo 1939, Claussen 1989, Claussen and Hauser 1990, Hurkmans 1993, Kassebeer 1995, 1998, 1999, 2000, Părvu et al. 2006, Popović et al. 2015, Acanski et al. 2016, van Steenis et al. 2017, Vujić et al. 2018, Ebejer et al. 2019).

Morocco has the highest number of total Syrphidae species recorded in the North Africa Mediterranean area so far (N = 150). Ninety-one species are reported from Algeria (Peck 1988, Dirickx 1994, Haffaressas et al. 2017), while 69, 51 and 34 species are recorded from Tunisia (Peck 1988, Claussen and Hauser 1990, Dirickx 1994), Egypt (Peck 1988, Dirickx 1994, El-Hawagry and Gilbert 2019) and Libya (Peck 1988, Dirickx 1994), respectively. Below, we provide an annotated checklist of all syrphid species recorded from Morocco.

Altogether 150 species are recorded in this work, two species are newly recorded for Morocco, and 24 others are new records for at least one of the Moroccan regions: Rif (10), Eastern region (4), High Atlas (5), Middle Atlas (5), and Anti Atlas (6). Morocco has 12 endemic species.

## Materials and methods

Syrphidae were collected from 79 localities across the main regions in Morocco, mostly in the Rif Mountains, including the Eastern region, the High Atlas, Middle Atlas and Anti Atlas. Additional material and databases indicated in the text are provided by other researchers.

Collecting was mainly carried out by sweep netting and Malaise trapping adults and/or rearing larvae and puparia in the laboratory from collected substrates in aquatic ecosystems following the technique used by Afzan and Belqat (2016). The majority of the Moroccan hoverflies were identified from external characteristics, except for some species for which male genital morphology was used to confirm their identity. Identifications were done with specific keys (Verlinden 1991, Kassebeer 1995a, Hauser and Kassebeer 1998, Kassebeer 1998b, Kassebeer 1999b, van Veen 2004, Marcos-García et al. 2007, Speight and Sarthou 2013). Fourty-six of the species were identified with the help of Bastiaan Wakkie (Brussels, Belgium).

**Figure 1.**
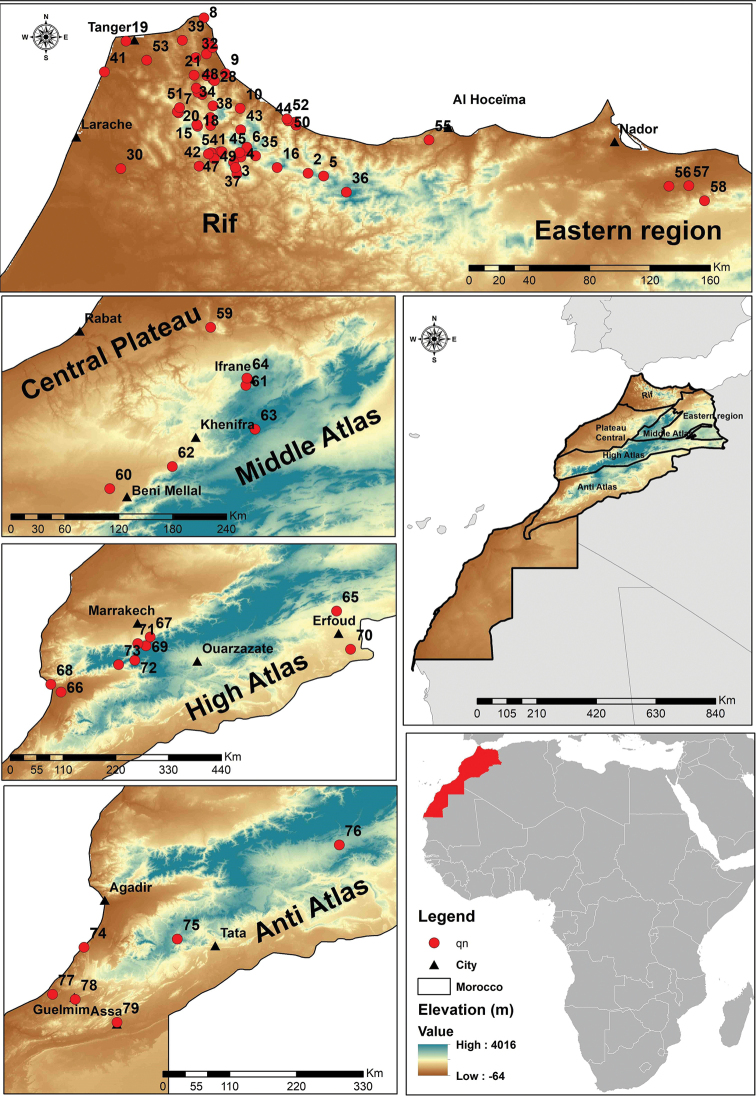
Map of Morocco indicating the collecting sites for hoverflies in this study.

A total of 690 Syrphidae specimens was collected from 79 Moroccan sites (Table 1, Fig. 1). Maps with collecting localities were created with ArcGIS (Geographic Information System). Literature references on the occurrence in Morocco are given for each species, together with the region of the country from which the species is known. Additional information is given in the section ’Comments’. The Distribution as it is given for individual species is based on Speight (2018) and Mengual et al. (2020), as the most up-to-date reference, although other published works were consulted for specific taxa in order to obtain a more accurate distribution.

**Table 1.** Sampling sites (in alphabetical order) with locality names, altitudes (meters), and geographical coordinates (latitude/longitude).

Station	Locality	Province	Altitude (m)	Geographical coordinates
**Rif**				
1. Aïn Afersiw	Mezine	Chefchaouen	750	35°05.945'N, 05°20.439'W
2. Aïn Boughaba	Jbel Bou Bessoui, Cedar Forest	Chefchaouen	1530	34°58.779'N, 04°46.366'W
3. Aïn El Ma Bared	Bouzthate	Chefchaouen	1270	35°00.333'N, 05°12.105'W
4. Aïn El Maounzil	National Park of Talassemtane	Chefchaouen	1110	35°04.577'N, 05°10.406'W
5. Aïn Quanquben	Jbel Bou Bessoui	Chefchaouen	1600	34°57.750'N, 04°40.783'W
6. Aïn Takhninjoute	Bab Rouida, National Park of Talassemtane	Chefchaouen	1510	35°06.881'N, 05°08.270'W
7. Aïn Sidi Brahim Ben Arrif	Road Moulay Abdessalam	Larache	900	35°20.398'N, 05°32.712'W
8. Belyounech	Jbel Moussa	M’diq Fnideq	60	35°54.389'N, 05°23.630'W
9. Bni Maaden	Bni Maaden	Tétouan	20	35°34.420'N, 05°15.901'W
10. Dam Entrasol	Ouedlaou Bni Ferten	Chefchaouen	40	35°22.015'N, 05°10.626'W
11. Daya El Birdiyel	Ânasar	Chefchaouen	1300	35°01.089'N, 04°59.477'W
12. Dam Moulay Bouchta	Larbaa Beni Hassan	Tétouan	360	35°15.864'N, 05°21.221'W
13. Dam Nakhla	Zinat	Tétouan	240	35°26.954'N, 05°24.326'W
14. Dam Smir	Bouzaghlale (M’diq)	Tétouan	20	35°41.491'N, 05°22.673'W
15. Daya Amsemlil	Jbel Bouhachem	Tétouan	1060	35°15.596'N, 05°25.917'W
16. Daya El Ânassar	Bab Berred	Chefchaouen	1180	35°00.788"N/04°57.419'W
17. Daya Jbel Zemzem	Jbel Zemzem	Tétouan	220	35°45.457'N, 05°22.189'W
18. Daya Mtahen	Jbel Bouhachem	Larache	970	35°16.195'N, 05°26.158'W
19. Daya Rahrah	Tanger	Tanger-Assilah	60	35°46.063'N, 05°51.517'W
20. Daya Tazia	Road Moulay Abdessalam	Larache	720	35°20.814'N, 05°33.139'W
21. Douar Dacheryène	Dacheryène	Tétouan	130	35°33.863'N, 05°27.162'W
22. Douar Kitane	Kitane	Tétouan	50	35°32.412'N, 05°20.393'W
23. Forest Bab El Karne	Tamakoute	Chefchaouen	1250	34°58.510'N, 05°11.838'W
24. Forest El Mahfoura	Zaouia Sidi Kacem, Amsa	Tétouan	800	35°27.724'N, 05°25.807'W
25. Forest house	National Park of Talassemtane	Chefchaouen	1670	35°08.076'N, 05°08.262'W
26. Garden Ksar Al Rimal	Kabila	M’diq-Fnideq	10	35°43.806'N, 05°20.509'W
27. Jumb Kitane	Kitane	Tétouan	40	35°32.759'N, 05°20.420'W
28. Halouma Kitane	Kitane	Tétouan	140	35°31.912'N, 05°19.861'W
29. 1 km after Derdara	Tanakoub	Chefchaouen	510	35°06.419'N, 05°17.938'W
30. Ksar El Kébir	Ksar El Kébir	Larache	25	35°00.441'N, 05°53.352'W
31. Meadow Mizoghar	El Khizana	Chefchaouen	1000	35°02.725'N, 05°12.969'W
32. Meadow Fahs Lmhar	Al Alyiene	M’diq Fnideq	590	35°40.148'N, 05°26.537'W
33. Oued Aârkoub	Aârkoub	Chefchaouen	140	35°15.943'N, 04°50.631'W
34. Oued Achekrade	Douar Aouzighen	Tétouan	640	35°22.931'N, 05°20.364'W
35. Oued Ametrasse	Chrafate	Chefchaouen,	830	35°05.014'N, 05°05.130'W
36. Oued Azila	Azila, Jbel Tidghine	Al Hoceïma	1600	34°52.028'N, 04°32.609'W
37. Oued 15 km from Fifi	Bouzthate	Chefchaouen	1260	35°00.805'N, 05°12.365'W
38. Oued Boumarouil	Larbaa Beni Hassen	Tétouan	550	35°18.733'N, 05°21.271'W
39. Oued Bin El Ouidane	Ksar Sghir	Tanger-Tétouan	90	35°46.356'N, 05°31.343'W
40. Oued Derdara	Derdara	Chefchaouen	400	35°06.484'N, 05°17.147'W
41. Oued Mharhar	Tanger	Tanger-Assilah	5	35°35.040'N, 05°59.220'W
42. Oued El Koub	Souk El Had	Chefchaouen	120	35°01.298'N, 05°25.333'W
43. Oued Farda	Akchour	Chefchaouen	400	35°14.350'N, 05°10.460'W
44. Oued Jnane Niche	Jnane Niche	Chefchaouen	40	35°17.019'N, 04°51.233'W
45. Oued Majjou (Hafa meqlouba)	Majjou village	Chefchaouen	800	35°06.175'N, 05°10.836'W
46. Oued Martil	Mhannech 2	Tétouan	9	35°33.693'N, 05°22.510'W
47. Oued Mezine	Mezine	Chefchaouen	780	35°06.104'N, 05°21.177'W
48. Oued Sahel	Ben Karrich	Tétouan	40	35°29.238'N, 05°26.352'W
49. Oued Sidi Ben Sâada	Derdara	Chefchaouen	220	35°03.921'N, 05°19.895'W
50. Oued Sidi Yahya Aârab	Sidi Yahya Aârab	Chefchaouen	60	35°17.545'N, 04°53.503'W
51. Oued Taïda	Taïda	Larache	500	35°22.250'N, 05°32.355'W
52. Stream at 1 km from Sidi Yahya Aârab	Sidi Yahya Aârab	Chefchaouen	2120	35°18.203'N, 04°53.965'W
53. Village Sebt Zinate	Village Sebt Zinate	Tanger-Assilah	60	35°39.272'N, 05°44.071'W
54. Tributary Oued Tazarine	Mezine	Chefchaouen	730	35°05.670'N, 05°21.991'W
55. Tributary Tarmast	National Park of Al Hoceïma	Al Hoceïma	170	35°10.666'N, 04°03.088'W
**Eastern region**				
56. Farm Saf-Saf	Douar Oueld Ben Amrou Aklim	Berkane	60	34°54.122'N, 02°37.212'W
57. Oued El Khemis	Road vers Aklim	Berkane	80	34°54.330'N, 02°30.097'W
58. Oued Tafoughalt	Tafoughalt, Beni Snassen	Berkane	750	34°48.941'N, 02°24.471'W
**Middle Atlas Mts**				
59. Aïn Ouilili	Sidi Moulay Zarhoun	Meknès	490	34°03.216'N, 05°31.050'W
60. Bridge Oued Oum Er-rabie (Douar Ahl Souss)	El Had Labradia	Fkih Bensalah	420	32°25.865'N, 06°31.883'W
61 Douar Ben Smim	Ben Smim	Ifrane	1690	33°28.200'N, 05°09.780'W
62. Douar Zaouiat Cheikh	Zaouiat Cheikh	Khénifra	700	32°39.246'N, 05°54.154'W
63. Oued d’Ifrane	Gorge of Zad	Ifrane	2140	33°01.740'N, 05°04.260'W
64. Vicinity of Ifrane	Ifrane	Ifrane	1660	33°32.580'N, 05°09.240'W
**High Atlas Mts**				
65. Aïn Zarka of Meski	Madkhal Meski	Errachidia	970	31°51.388'N, 04°16.917'W
66. Agadir airport	Ikhourbane	Agadir	50	30°20.400'N, 09°26.820'W
67. Douar Akhlij Tnine Ourika	Ourika	Marrakech	870	31°22.385'N, 07°46.608'W
68. Douar Aourir	Aouirir	Agadir	200	30°28.980'N, 09°38.340'W
69. Lac Oukaïmeden	Oukaïmeden	Marrakech	2590	31°12.420'N, 07°51.120'W
70. Palm grove Igrane	Merzouga village Hassilalbied	Errachidia	710	31°08.520'N, 04°01.260'W
71. Vicinity of Asni	Asni	Marrakech	1400	31°14.880'N, 08°00.480'W
72. Vicinity of Ijoukak	Ijoukak	Marrakech	1790	30°56.100'N, 08°03.840'W
73. Tizi N’Test	Tizi N’Test	Taroudante	1850	30°51.180'N, 08°22.080'W
**Anti Atlas Mts**				
74. Beach of Tamellalt	Tamellalt	Tiznit	70	29°43.080'N, 09°55.020'W
75. Douar Issafen	Issafen	Tata	1110	29°50.460'N, 08°32.100'W
76. Douar Zaouia	Kelâa M’gouna	Ouarzazate	1240	31°14.275'N, 06°08.169'W
77. 1 km before Douar Aïn Lahmar	Jbel Ras Tarf	Guelmim	190	29°01.320'N, 10°23.100'W
78. Msidira	El Filaha	Guelmim	260	28°56.865'N, 10°02.979'W
79. Oued Assa	Assa	Assa zag	310	28°36.507'N, 09°25.800'W

Nomenclature followed Speight (2018) and subfamily classification followed Mengual et al. (2015). Synonyms are given as far as they have been used in the literature dealing with the Moroccan fauna. All the specimens collected in this study are deposited in the collection of Diptera of the Department of Biology, Faculty of Sciences, University Abdelmalek Essaâdi, Tétouan.

## Results

### Subfamily SYRPHINAE Latreille, 1802

#### Tribe MELANOSTOMINI Williston, 1885

##### Genus *MELANOSTOMA* Schiner, 1860

***Melanostoma mellinum* (Linnaeus, 1758)**

**Literature records.** Rif (Becker and Stein 1913: 88, Gil Collado 1929: 405, Kanervo 1939: 2–3). Central Plateau (Séguy 1934: 162). Middle Atlas (Kanervo 1939, Timon-David 1951: 145, Leclercq 1961: 241, Claussen and Hauser 1990: 435). High Atlas (Kanervo 1939, Timon-David 1951, Claussen 1989b: 359, 373, Claussen and Hauser 1990). Distribution map Dirickx (1994: 76, 218).

**New sites.** Rif: Daya Rahrah, 8/IV/2013, 4♂♂; Aïn El Ma Bared, 06/V/2013, 4♀♀, 06/V/2014, 2♂♂; Oued Derdara, 24/V/2013, 1♀; Daya El Ânassar, 24/V/2013, 1♂; Daya Mtahen, 30/V/2013, 1♂; Garden Ksar Al Rimal, 26/V/2013, 7♂♂, 17♀♀, 5/VI/2013, 1♂; Tributary Oued Tazarine, 11/VI/2013, 1♂; Oued Farda, 23/VI/2013, 1♂; Daya El Birdiyel, 27/VI/2013, 1♀; Forest house, 18/V/2014, 1♂, 1♀; Stream at 1 km from Sidi Yahya Aârab, 25/IV/2015, 1♂; Daya Amsemlil, 7/V/2015, 3♀♀, sweep net, leg. Sahib and Belqat. Eastern region: Farm Saf-Saf, 14/VI/2013, 3♂♂, 21♀♀, sweep net, leg. Sahib and Belqat. Middle Atlas: Oued d’Ifrane, 10/VI/2014, 4♂♂, sweep net, leg. Bot. High Atlas: Lac Oukaïmeden, 18/VI/2014, 1♂, 1♀, sweep net, leg. Bot.

**Distribution.** Holarctic.

**Comment.** New record for the Eastern Region.

***Melanostoma scalare* (Fabricius, 1794)**

**Literature records.** Rif (Gil Collado 1929: 405). Listed (Claussen 1989b: 373). Distribution map (Dirickx 1994: 76, 219).

**New sites.** Rif: Aïn El Ma Bared, 06/V/2013, 11♀♀; Oued Mezine, 11/VI/2013, 1♀; Aïn Quanquben, 27/VI/2013, 6♀♀; Aïn Takhninjoute, 17/V/2014, 1♂; Oued El Koub, 30/V/2014, 1♀, sweep net, leg. Sahib and Belqat.

**Distribution.** Palaearctic, Afrotropical, and Indomalayan regions.

##### Genus *PLATYCHEIRUS* Le Peletier and Serville, 1828

***Platycheirus ambiguus* (Fallén, 1817)**

**Literature record.** High Atlas (Kassebeer 1998b: 27).

**Distribution.** Palaearctic.

***Platycheirus atlasi* Kassebeer, 1998**

**Literature records.** Middle Atlas (Kassebeer 1998b: 28). High Atlas (Kassebeer 1998b: 28).

**Comment.** Endemic to Morocco.

**Distribution.** Morocco.

***Platycheirus fulviventris* (Macquart, 1829)**

**Literature record.** High Atlas (Ebejer et al. 2019: 150).

**Distribution.** Palaearctic.

***Platycheirus manicatus* (Meigen, 1822)**

**Literature records.** High Atlas (Séguy 1961: 74). Listed (Claussen 1989b: 373). Distribution map (Dirickx 1994: 111, 268).

**Distribution.** Palaearctic, Greenland, and Alaska.

***Platycheirus marokkanus* Kassebeer, 1998**

**Literature record.** High Atlas, Middle Atlas (Kassebeer 1998b: 31). Cited (Speight 2018: 209).

**New site.** Rif: Aïn Takhninjoute, 17/V/2014, 1♂. High Atlas: Douar Akhlij Tnine Ourika 18/IV/2015, 1♂, sweep net, leg. Sahib and Belqat.

**Comment.** New record for the Rif.

**Distribution.** Morocco and Portugal.

##### Genus *XANTHANDRUS* Verrall, 1901

***Xanthandrus comtus* (Harris, 1780)**

**Literature records.** Rif (Gil Collado 1929: 405). Listed (Claussen 1989b: 374). Distribution map (Dirickx1994: 128, 290).

**Distribution.** Palaearctic.

#### Tribe PARAGINI Glumac, 1961

##### Genus *PARAGUS* Latreille, 1804

***Paragus albifrons* (Fallén, 1817)**

**Literature records.** Middle Atlas (Kanervo1939: 2). Listed (Claussen 1989b: 373). Distribution map (Dirickx 1994: 98, 249).

**Distribution.** Palaearctic.

***Paragus atlasi* Claussen, 1989**

**Literature records.** High Atlas (Claussen 1989b: 359–361). Cited (Dirickx 1994: 99, Speight 2018: 175).

**Comment.** Endemic to Morocco.

**Distribution.** Morocco.

***Paragus bicolor* (Fabricius, 1794)**

**Literature records.** Rif (Becker and Stein 1913: 88). Central Plateau (Kanervo 1939: 2). Middle Atlas (Séguy 1930: 128, Kanervo 1939, Timon-David 1951: 144, Claussen 1989, Claussen and Hauser 1990). High Atlas (Kanervo 1939, Claussen 1989b: 361, 373, Claussen and Hauser 1990: 435). Anti Atlas (Séguy 1949: 156). Distribution map (Dirickx 1994: 98, 250).

**New sites.** Rif: Douar Dacheryène, 4/IV/2014, 1♀, sweep net, leg. Sahib and Belqat. High Atlas: Vicinity of Asni, 05/VI/2014, 1♀, sweep net, leg. Bot.

**Distribution.** Holarctic.

***Paragus cinctus* Schiner and Egger, 1853**

**Literature records.** High Atlas (Claussen 1989b: 361, 373). Cited (Speight 2018: 176). Distribution map (Dirickx 1994: 99, 250).

**New site.** High Atlas: Vicinity of Asni, 05/VI/2014, 1♂, sweep net, leg. Bot.

**Distribution.** Western and Central Palaearctic.

***Paragus coadunatus* Rondani, 1847**

**Literature records.** Middle Atlas (Claussen and Hauser 1990: 436). Cited (Speight 2018: 176). Distribution map (Dirickx 1994: 99, 251).

**New site.** Rif: Oued Mharhar, 16/VI/2009, 1♂, sweep net, leg. Bot.

**Comment.** New record from the Rif.

**Distribution.** Balearic Is, Canary Is, Sicily, Malta, Madeira, Turkey, and Morocco.

***Paragus flammeus* Goeldlin, 1971**

**Literature records.** Middle Atlas (Claussen and Hauser 1990: 435). Cited (Speight 2018: 178). Distribution map (Dirickx 1994: 100, 252).

**Distribution.** Western and Central Palaearctic.

***Paragus haemorrhous* Meigen, 1822**

**Literature records.** Recorded and listed from the Middle Atlas (Claussen 1989b: 359, 372). Distribution map (Dirickx 1994: 100, 252).

**Distribution.** Holarctic and Afrotropical regions.

***Paragus hermonensis* Kaplan, 1981**

**Literature record.** Middle Atlas (Claussen 1989b: 361, 373). Distribution map (Dirickx 1994: 100, 101, 252).

**Distribution.** Mediterranean Basin.

***Paragus pecchiolii* Rondani, 1857**

**Literature records.** Middle Atlas (Claussen and Hauser 1990: 435). Distribution map (Dirickx 1994: 101, 253).

**Distribution.** Western Palaearctic.

***Paragus quadrifasciatus* Meigen, 1822**

**Literature records.** Central Plateau (Claussen 1989b: 361, 373). Middle Atlas (Claussen and Hauser 1990: 435, Timon-David 1951: 144, as *Paragus pulcherrimus*). Distribution map (Dirickx 1994: 101, 253).

**New sites.** Rif: Douar Kitane, 19/VI/2014, 1♀, sweep net, leg. Sahib and Belqat. High Atlas: Agadir airport, 7/IV/2015, 1♀, leg. Schmid-Egger.

**Comment.** New record from the Rif and the High Atlas.

**Distribution.** Palaearctic.

***Paragus strigatus* Meigen, 1822**

**Literature records.** Central Plateau (Wiedemann 1824: 33, as *Paragus bimaculatus*). Middle Atlas (Claussen 1989b: 361, 373, Claussen and Hauser 1990: 435). High Atlas (Claussen 1989b: 361, 373). Cited (Speight 2018: 181). Distribution map (Dirickx 1994: 102, 254).

**Distribution.** Palaearctic.

***Paragus lis* (Fallén, 1817)**

**Literature records.** Rif (Becker and Stein 1913: 88, Leclercq 1961: 241, as *Paragus tibialis meridionalis*). Middle Atlas (Kanervo 1939: 2, Leclercq 1961: 241). High Atlas (Gil Collado 1929: 403, 404 (as Paragus tibialis var. meridionalis), Kanervo 1939: 2). Anti Atlas (Séguy 1949: 156). Listed (Claussen 1989b: 359, 373). Distribution map (Dirickx 1994: 102, 254).

**New sites.** High Atlas: Vicinity of Asni, 05/VI/2014, 1♂, 07/VI/2014, 2♂♂; Vicinity of Ijoukak, 17/VI/2014, 1♂, sweep net, leg. Bot.

**Distribution.** Western Palaearctic.

***Paragus vandergooti* Marcos-Garcia, 1986**

**Literature records.** Middle Atlas (Claussen and Hauser 1990: 435). High Atlas (Claussen 1989b: 362, 373). Cited (Speight 2018: 181). Distribution map (Dirickx 1994: 102, 255).

**Distibution.** France, Portugal, Spain, and Morocco.

#### Tribe CHRYSOTOXINI Newman, 1834

##### Genus *CHRYSOTOXUM* Meigen, 1803

***Chrysotoxum intermedium* (Meigen, 1822)**

**Literature records.** Rif (Becker and Stein 1913: 86, Gil Collado 1929: 414). Central Plateau (Părvu et al. 2006: 275). Middle Atlas (Séguy 1930: 131, 1961: 120). High Atlas (Séguy 1930: 131, Kanervo 1939: 4, Gil Collado 1929: 414). Listed (Claussen 1989b: 372). Distribution map (Dirickx 1994: 47, 178).

**New sites.** Rif: 1 km after Derdara, 11/VI/2013, 1♀; Oued Azila, 27/VI/2013, 1♂; Daya Jbel Zemzem, 23/IV/2014, 1♂; Aïn El Maounzil, 17/V/2014, 1♀, sweep net, leg. Sahib and Belqat. Eastern region: Oued Tafoughalt, 25/XI/2014, 1♂, sweep net, leg. Sahib and Belqat. Middle Atlas: Douar Zaouiat Cheikh, 19/III/2008, 2♂♂, 1♀, sweep net, leg. A. van Eck. High Atlas: Vicinity of Asni, 6/VI/2014, 2♀♀, sweep net, leg. Bot. Anti Atlas: Douar Issafen, 14/VI/2015, 3♀♀, 1♂, sweep net, leg. Schmid-Egger.

**Comment.** New record from the Eastern region and the Anti Atlas.

**Distribution.** Europe and Morocco.

***Chrysotoxum volaticum* Séguy, 1961**

**Literature records.** Middle Atlas (Timon-David 1951: 146, Leclercq 1961: 242, Séguy 1961: 123). High Atlas (Timon-David 1951, Claussen 1989b: 357, 372). Listed (Claussen 1989b: 372). Distribution map (Dirickx1994: 46, 49, 175, 181).

**Distribution.** Algeria and Morocco.

#### Tribe SYRPHINI Latreille, 1802

##### Genus *DASYSYRPHUS* Enderlein, 1838

***Dasysyrphus albostriatus* (Fallén, 1817)**

**Literature record.** High Atlas (Kassebeer 1998a: 22).

**Distribution.** Palaearctic.

##### Genus *EPISTROPHE* Walker, 1852

***Epistrophe eligans* (Harris, 1780)**

**Literature records.** Rif (Becker and Stein 1913: 88, as *Syrphus ochrostoma*). Middle Atlas (Kassebeer 1998a: 22). High Atlas (Kassebeer 1998a: 22). Listed (Claussen 1989b: 372, as *Epistrophe ochrostoma*).

**New site.** Rif: Daya Tazia, 25/VI/2014, 1♂, sweep net, leg. Sahib and Belqat.

**Distribution.** Western Palearctic.

##### Genus *EPISYRPHUS>* Matsumura and Adachi, 1917

***Episyrphus balteatus* (De Geer, 1776)**

**Literature records.** Rif (Becker and Stein 1913: 88, as *Syrphus balteatus*, Gil Collado 1929: 406, Kanervo 1939: 3, as *Epistrophe balteata*). Central Plateau (Séguy 1930: 129, as *Syrphus balteatus*, Timon-David 1951: 144, as *Epistrophe balteatus*). High Atlas (Séguy 1930, Timon-David 1951). Listed (Claussen 1989b: 372). Distribution map (Dirickx 1994: 53, 189).

**New sites.** Rif: Village Sebt Zinate, 15/III/2008, 1♀, sweep net, leg. AvEck; Aïn Sidi Brahim Ben Arrif, 17/IV/2013, 1♂; Dam Nakhla, 24/V/2013, 1♂, 1♀; Aïn Boughaba, 24/V/2013, 1♀; Garden Ksar Al Rimal, 5/VI/2013, 1♀; Oued Aârkoub, 19/IV/2013, 4♀♀; Daya Rahrah, 2/XII/2013, 1♂, 1♀; Oued Sahel, 5/III/2014, 1♀; Dam Moulay Bouchta, 5/III/2014, 26♂♂; Forest house, 17/VI/2014, 1♂; Ksar El Kébir, 19/IV/2014, 1♂, 1♀; Daya Jbel Zemzem, 23/IV/2014, 13♂♂, 27/IV/2014, 1♀; Oued Taïda, 12/III/2015, 1♂; Stream at 1 km from Sidi Yahya Aârab, 25/IV/2015, 1♂; Aïn Quanquben, 25/IV/2015, 1♀; Oued Majjou (Hafa meqlouba), 25/XII/2015, 1♀; Forest Bab El Karne, 25/XII/2015, 1♂; Douar Kitane, 23/I/2016, 2♂♂, 1♀, sweep net, leg. Sahib and Belqat; Douar Kitane, 7/IV/2017, 54♂♂, 14♀♀, malaise trap, leg. Sahib and Belqat; Forest El Mahfoura, 13/V/2018, 18♂♂, 4♀♀, sweep net, leg. Sahib and Belqat. High Atlas: Aïn Zarka of Meski, 20/III/2008, 1♂, sweep net, leg. A. van Eck. Anti Atlas: Douar Zaouia, 11/VI/2015, 1♂, sweep net, leg. Sahib and Belqat.

**Distribution.** Palaearctic and Indomalayan regions.

##### Genus *EUPEODES* Osten Sacken, 1877

***Eupeodes corollae* (Fabricius, 1794)**

**Literature records.** Rif (Becker and Stein 1913: 88, as *Syrphus corollae*, Kanervo 1939: 3, as *Syrphus corollae*, Gil Collado 1929: 406, as *Syrphus corollae*). Central Plateau (Timon David 1951: 144, as *Syrphus corollae*, Gil Collado 1929: 406). Middle Atlas (Leclercq 1961: 241, as *Syrphus corollae*). High Atlas (Séguy 1930, Timon-David 1951). Anti Atlas (Timon David 1951). Listed (Bigot 1884: 88, as *Syrphus berber*, Séguy 1930: 129, 1961: 103, Claussen 1989b: 372). Distribution map (Dirickx 1994: 89, 235 (as *Metasyrphus corollae*)).

**New site.** Rif: Village Sebt Zinate, 15/III/2008, 1♀, sweep net leg. AvEck; Aïn El Ma Bared, 27/IV/2013, 1♀; Garden Ksar Al Rimal, 20/V/2013, 1♀; Oued Bin EL Ouidane, 14/XI/2013, 1♀; Oued Sahel, 5/III/2014, 1♂; Aïn Takhninjoute, 17/V/2014, 31♂♂; Stream at 1 km from Sidi Yahya Aârab, 25/IV/2015, 2♂♂, 3♀♀; Oued Jnane Niche, 16/III/2014, 1♂, 25/IV/2015, 1♂, 1♀; Dam Smir, 27/IV/2014, 1♂; Oued Boumarouil, 10/V/2014, 1♂; Daya Jbel Zemzem, 23/IV/2014, 1♂, 3♀♀, 17/IV/2014, 6♂♂, 3♀♀; Oued Majjou (Hafa meqlouba), 10/V/2014, 1♂, 27/IV/2015, 1♂; Meadow Fahs Lmhar, 15/IV/2018, 1♀, sweep net, leg. Sahib and Belqat. Douar Kitane, 21/IV/2018, 1♂, reared by Sahib and Belqat; Forest El Mahfoura, 13/V/2018, 12♂♂, 7♀♀, sweep net, leg. Sahib and Belqat. Middle Atlas: Douar Zaouiat Cheikh, 19/III/2008, 1♀, sweep net, leg. A. van Eck. High Atlas: Vicinity of Asni, 6/VI/2014, 1♀; Vicinity of Ijoukak, 17/VI/2014, 1♀, sweep net, leg. Bot; Lac Oukaïmeden, 18/VI/2014, 1♂, sweep net, leg. Bot.

**Distribution.** Palaearctic, Afrotropical Region, and Taiwan.

***Eupeodes latifasciatus* (Macquart, 1829)**

**Literature records.** Anti Atlas (Séguy 1949: 156, Séguy 1953: 84, as *Syrphus latifasciatus*). Listed (Claussen 1989b: 372). Distribution map (Dirickx 1994: 89, 236 (as *Metasyrphus latifasciatus*)).

**New sites.** Rif: Douar Kitane, 2/V/2014, 1♂, 2♀♀; Forest house, 18/V/2014, 1♂; Oued Ametrasse, 27/IV/2015, 1♂, sweep net, leg. Sahib and Belqat.

**Comment**. New record for the Rif.

**Distribution.** Holarctic and India.

***Eupeodes luniger* (Meigen, 1822)**

**Literature records.** Central Plateau (Gil Collado 1929: 406, as *Syrphus luniger*. Listed (Claussen 1989b: 372). Distribution map (Dirickx 1994: 90, 237 (as *Metasyrphus luniger*)).

**New sites.** Rif: Oued Majjou (Hafa meqlouba), 10/V/2014, 3♂♂; Oued Martil, 13/XII/2013, 1♀; Belyounech, 9/VI/2013, 1♀, sweep net, leg. Sahib and Belqat; Douar Kitane, 24/IV/2017, 1♀, malaise trap, leg. Sahib and Belqat. Middle Atlas: Douar Ben Smim, 08/VI/2014, 1♂, sweep net, leg. Bot. High Atlas: Vicinity of Asni, 6/VI/2014, 1♀, sweep net, leg. Bot.

**Comments.** New records from the Rif, the Middle Atlas and the High Atlas.

**Distribution.** Palaearctic and northern India.

***Eupeodes nuba* (Wiedemann, 1830)**

**Literature records.** High Atlas (Claussen 1989b: 359, 372). Cited (Bigot 1884: 88, as *Syrphus rufinasutus*, Séguy 1961: 107, as *Syrphus rufinasutus*, Speight 2018: 108). Distribution map (Dirickx 1994: 90, 238 (as *Metasyrphus nuba*)).

**Distribution.** Palaearctic.

##### Genus *ISCHIODON* Sack, 1913

***Ischiodon aegyptius* (Wiedemann, 1830)**

**Literature records.** Central Plateau (Gil Collado 1929: 406, Timon-David 1951: 145). Anti Atlas (Timon-David 1951).

**Distribution.** Afrotropical Region, Mediterranean Basin, and Canary Islands.

##### Genus *LAPPOSYRPHUS* Dusek and Laska 1967

***Lapposyrphus lapponicus* (Zetterstedt, 1838)**

**Literature records.** Rif (Becker and Stein 1913: 88, as *Syrphus arcuatus*). Listed (Claussen 1989b: 372, as *Eupeodes lapponicus*). Distribution map (Dirickx 1994: 89, 236 (as *Metasyrphus lapponicus*)).

**Distribution.** Palaearctic, northern America from Alaska to California.

##### Genus *MELISCAEVA* Frey, 1946

***Meliscaeva auricollis* (Meigen, 1822)**

**Literature records.** Rif (Becker and Stein 1913: 89, as *Syrphus auricollis*, Gil Collado 1929: 406, as *Syrphus auricollis*). Central Plateau (Timon-David 1951: 145, as *Epistrophe auricollis*). Listed (Claussen 1989b: 373). Distribution map (Dirickx 1994: 76, 219).

**New sites.** Rif: Aïn El Ma Bared, 27/IV/2013, 1♀; Daya El Ânassar, 24/V/2013, 1♂; Belyounech, 9/VI/2013, 1♀; Oued Mezine, 11/VI/2013, 1♂; Dam Moulay Bouchta, 5/IV/2014, 1♀; Aïn Afersiw, 11/VI/2013, 3 ♂♂, 7♀♀; Dam Entrasol, 14/XI/2014, 1♀; Oued 15 km from Fifi, 6/V/2014, 1♀; Jumb Kitane, 10/III/2015, 1♀; Oued Majjou (Hafa meqlouba), 10/V/2014, 1♀; Douar Kitane, 2/II/2015, 1♂; Oued Sahel, 5/IV/2014, 1♂. Middle Atlas: Aïn Ouilili, 18/II/2016, 2♀♀, sweep net, leg. Sahib and Belqat.

**Comment**. New record for the Middle Atlas.

**Distribution.** Western Palaearctic.

***Meliscaeva cinctella* (Zetterstedt, 1843)**

**Literature records.** Middle Atlas (Séguy 1934: 161, as *Syrphus cinctellus*). Listed (Claussen 1989b: 373). Distribution map (Dirickx 1994: 77, 219).

**Distribution.** Holarctic.

##### Genus *SCAEVA* Fabricius, 1805

***Scaeva albomaculata* (Macquart, 1842)**

**Literature records.** Rif (Gil Collado 1929: 405, as *Lasiopticus albomaculata*). Central Plateau (Timon-David 1951: 145, as *Lasiopticus albomaculata*). Middle Atlas (Leclercq 1961: 241, Claussen 1989b: 359). Listed (Séguy 1961: 94, Claussen 1989b: 373). Cited (Speight 2018: 225). Distribution map (Dirickx 1994: 115, 275).

**New sites.** Rif: Belyounech, 9/VI/2013, 1♂, sweep net, leg. Sahib and Belqat. High Atlas: Aïn Zarka of Meski, 20/III/2008, 1♀, sweep net, leg. AvEck; Lac Oukaïmeden, 19/VI/2014, 1♀, sweep net, leg. Bot.

**Comment.** New records for the High Atlas.

**Distribution.** Palaearctic.

***Scaeva dignota* (Rondani, 1857)**

**Literature records.** Middle Atlas (Claussen and Hauser 1990: 436). Distribution map (Dirickx 1994: 116, 275).

**New sites.** Rif: Oued Majjou (Hafa meqlouba), 27/IV/2015, 1♀; Daya Mtahen, 26/III/2016, 1♂, sweep net, leg. Sahib and Belqat.

**Comment.** New records for the Rif.

**Distribution.** Western Palaearctic.

***Scaeva mecogramma* (Bigot, 1860)**

**Literature records.** Rif (Kassebeer1998a: 23). Middle Atlas (Kassebeer1998a: 23). High Atlas (Kassebeer1998a: 23).

**Distribution.** Spain, Southern France, Corsica, Italy, Switzerland, and Morocco.

***Scaeva pyrastri* (Linnaeus, 1758)**

**Literature records.** Rif (Becker and Stein 1913: 88, as *Catabomba pyrastri*, Gil Collado 1929: 405). Central Plateau (Gil Collado 1929: 405, as *Lasiopticus pyrastri*, Timon-David 1951: 145, as *Lasiopticus pyrastri*). Middle Atlas (Séguy 1930: 128, as *Lasiopticus pyrastri*, Timon-David 1951: 145). Listed (Claussen 1989b: 373). Distribution map (Dirickx 1994: 116, 276).

**New sites.** Rif: Daya Jbel Zemzem, 23/IV/2014, 1♀; Stream at 1 km from Oued Sidi Yahya Aârab, 25/IV/2015, 1♀, sweep net, leg. Sahib and Belqat. Anti Atlas: 1 km before Douar Aïn Lahmar, 10/IV/2015, 1♀, sweep net, leg. Schmid-Egger.

**Comment.** New record for the Anti Atlas.

**Distribution.** Holarctic and India.

***Scaeva selenitica* (Meigen, 1822)**

**Literature records.** High Atlas (Séguy 1930: 128, as *Lasiopticus selenitica*). Listed (Claussen 1989b: 373). Distribution map (Dirickx 1994: 116, 276).

**Distribution.** Palaearctic.

##### Genus *SPHAEROPHORIA* Lepeletier and Serville, 1828

***Sphaerophoria interrupta* (Fabricius, 1805)**

**Literature records.** Rif (Becker and Stein 1913: 87, as *Sphaerophoria menthastri*, Kanervo 1939: 3). Central Plateau (Kanervo 1939: 3, as *Sphaerophoria menthastri*, Timon-David 1951: 145, as *Sphaerophoria menthastri*). Middle Atlas (Kanervo 1939, Timon-David 1951). High Atlas (Kanervo 1939). Anti Atlas (Timon-David 1951). Cited (Séguy 1961: 109, as *Sphaerophoria menthastri*). Listed (Claussen 1989b: 373). Distribution map (Dirickx 1994: 118).

**Comment.***S. interrupta* records in Morocco need confirmation.

**Distribution.** Western Palaearctic and Siberia.

***Sphaerophoria rueppelli* (Wiedemann, 1830)**

**Literature records.** Rif (Kanervo 1939: 4). Central Plateau (Timon-David 1951: 145, Kanervo 1939). High Atlas (Kanervo 1939, Timon-David 1951, Claussen 1989b). Anti Atlas (Timon-David 1951). Cited (Séguy 1961: 110). Listed (Claussen 1989b: 359, 373). Distribution map (Dirickx 1994: 119, 278).

**New sites.** Rif: Tributary Tarmast, 26/VI/2013, 1♂; Oued Sidi Ben Sâada, 6/V/2015, 2♂♂; Daya Amsemlil, 7/V/2015, 1♂. Eastern region: Farm Saf-Saf, 14/VI/2013, 4♂♂, sweep net, leg. Sahib and Belqat. Middle Atlas: Oued d’Ifrane: 10/VI/2014, 1♂, sweep net, leg. Bot.

**Comment.** New records for the Eastern region and the Middle Atlas.

**Distribution.** Palaearctic and Afrotropical regions.

***Sphaerophoria scripta* (Linnaeus, 1758)**

**Literature records.** Rif (Becker and Stein 1913: 87, Gil Collado 1929: 406, Kanervo 1939: 4). Central Plateau (Kanervo 1939, Timon-David 1951: 145). Middle Atlas (Séguy 1930: 129, Kanervo 1939, Timon-David 1951, Leclercq 1961: 241, Claussen 1989). High Atlas (Kanervo 1939, Claussen 1989b: 359). Listed (Claussen 1989b: 359, 373). Distribution map (Dirickx 1994: 119, 278).

**New sites.** Rif: Oued Aârkoub, 19/IV/2013, 1♂; Dam Nakhla, 25/IV/2013, 1♀; Meadow Mizoghar, 27/IV/2013, 1♂, 1♀; Oued Derdara, 24/V/2013, 3♀♀; 1 km after Derdara, 11/VI/2013, 1♂, 1♀; Daya El Birdiyel, 27/VI/2013, 8♀♀, sweep net, leg. Sahib and Belqat; Palm grove Igrane, 12/VI/2014, 2♂♂, sweep net, leg. Bot; Aïn Quanquben, 27/VI/2013, 7♂♂, 7♀♀; Forest house, 18/V/2014, 1♂; Oued Sidi Ben Sâada, 6/V/2015, 1♂, 1♀; Daya Mtahen, 7/V/2015, 1♂; Daya Amsemlil, 7/V/2015, 1♂, 1♀; Forest El Mahfoura, 13/V/2018, 2♂♂, sweep net, leg. Sahib and Belqat. Eastern region: Farm Saf-Saf, 14/VI/2013, 47♂♂, 53♀♀, sweep net, leg. Sahib and Belqat. Middle Atlas: Oued d’Ifrane, 10/VI/2014, 2♂♂, sweep net, leg. Bot. High Atlas: Vicinity of Asni, 07/VI/2014, 2♂♂, sweep net, leg. Bot; Lac Oukaïmeden, 18/VI/2014, 1♂, sweep net, leg. Bot. Anti Atlas: Msidira, 18/V/2015, 1♂, 2♀♀, sweep net, leg. Sahib and Belqat.

**Comment**. New records for the Eastern region and the Anti Atlas.

**Distribution.** Palaearctic, Greenland, Nepal, and Kashmir.

***Sphaerophoria taeniata* (Meigen, 1822)**

**Literature records.** Central Plateau (Timon-David 1951: 10, as Sphaerophoria menthastri var. taeniata). Listed (Claussen 1989b: 373). Distribution map (Dirickx 1994: 119, 279).

**Distribution.** Palaearctic.

##### Genus *SYRPHUS* Fabricius, 1775

***Syrphus ribesii* (Linnaeus, 1758)**

**Literature records.** Rif (Kassebeer 1998a: 23).

**New site.** Rif: Douar Kitane, 30/I/2014, 1♀, sweep net, leg. Sahib and Belqat.

**Distribution.** Holarctic.

***Syrphus vitripennis* Meigen, 1822**

**Literature records.** Rif (Gil Collado 1929: 406). Listed (Claussen 1989b: 374). Distribution map (Dirickx 1994: 125, 286).

**Distribution.** Palaearctic, western North America, and Taiwan.

##### Genus *XANTHOGRAMMA* Schiner, 1860

***Xanthogramma dives* (Rondani, 1857)**

**Literature record.** High Atlas (Ebejer et al. 2019)

**Distribution.** Europe and Morocco.

***Xanthogramma evanescens* Becker, 1913**

**Literature records.** Rif (Becker and Stein 1913: 87). Listed (Claussen 1989b: 374). Distribution map (Dirickx 1994: 128).

**Comment.** Endemic to Morocco.

**Distribution.** Morocco.

***Xanthogramma marginale*** (**Loew, 1854)**

**Literature records.** Rif (Becker and Stein 1913: 86, as Xanthogramma marginale var. morenae), Gil Collado 1929: 406, as Xanthogramma marginale var. morenae), Kanervo 1939: 4). High Atlas (Kanervo 1939). Middle Atlas (Claussen and Hauser 1990: 436). Cited (Séguy 1961: 113, Speight 2018: 258). Listed (Claussen 1989b: 374). Distribution map (Dirickx 1994: 129, 291).

**New sites.** Rif: Village Sebt Zinate, 15/III/2008, 1♀, sweep net, leg. AvEck; Oued Majjou (Hafa meqlouba), 10/V/2014, 1♂; Oued Ametrasse, 18/IV/2015, 1♂, sweep net, leg. Sahib and Belqat; Douar Kitane, 7/IV/2017, 1♂, malaise trap, leg. Sahib and Belqat.

**Distribution.** Western part of the Mediterranean Basin.

***Xanthogramma pedissequum* (Harris, 1776)**

**Literature records.** Rif (Gil Collado 1929: 406, as *Xanthogramma ornatum*). Listed (Claussen 1989b: 374). Distribution map (Dirickx 1994: 129, 292).

**Comment.***X. pedissequum* records in Morocco need confirmation.

**Distribution.** Europe and Morocco.

Subfamily ERISTALINAE Rondani, 1845

#### Tribe CALLICERINI Brues and Melander, 1932

##### Genus *CALLICERA* Panzer, 1806

***Callicera fagesii* Guérin-Méneville, 1844**

**Literature record.** High Atlas (Kassebber 1998a: 22).

**Distribution.** Europe and Morocco.

***Callicera rufa* Schummel, 1842**

**Literature records.** Rif (Gil Collado 1929: 414). Listed (Claussen 1989b: 372). Distribution map (Dirickx 1994: 23, 139).

**Distribution.** Western Palaearctic.

#### Tribe CERIOIDINI Wahlgren, 1909

##### Genus *CERIANA* Rafinesque, 1815

***Ceriana conopsoides* (Linnaeus, 1758)**

**Literature records.** Middle Atlas (Séguy 1930: 131, as *Cerioides conopsoides*). Listed (Claussen 1989b: 372). Distribution map (Dirickx 1994: 23, 140).

**Distribution.** Palaearctic.

***Ceriana vespiformis* (Latreille, 1804)**

**Literature records.** Rif (Becker and Stein 1913: 88, as *Cerioides vespiformis*, Gil Collado 1929: 414). Central Plateau (Séguy 1930: 131, as *Cerioides vespiformis*, Leclercq 1961). Middle Atlas (Séguy 1930, Kanervo 1939: 5, Leclercq 1961: 242, as *Cerioides vespiformis*). High Atlas (Gil Collado 1929, as *Cerioides vespiformis*, Kanervo 1939, Claussen 1989b: 365, 372). Cited (Séguy 1961: 210, Speight 2018: 21). Distribution map (Dirickx 1994: 23, 140).

**New sites.** Rif: 1 km after Derdara, 11/VI/2013, 1♂; Meadow Mizoghar, 6/V/2014, 1♂; Oued Achekrade, 31/V/2014, 1♂, sweep net, leg. Sahib and Belqat.

**Distribution.** Western Palaearctic.

#### Tribe CHEILOSINI Williston, 1885

##### Genus *CHEILOSIA* Meigen, 1822

***Cheilosia brunnipennis* Becker, 1894**

**Literature records.** High Atlas (Kanervo 1939: 2, as *Chilosia flavipes*, Kassebeer 1998c: 58, 59). Cited (Séguy 1961: 38, 39, Speight 2018: 29). Listed (Claussen 1989b: 372, as *Cheilosia flavipes*).

**Distribution.** Western Palaearctic.

***Cheilosia grossa* (Fallén, 1817)**

**Literature record.** Hight Atlas (Kassebeer 1998c: 59). Cited (Speight 2018: 35).

**Distribution.** Palaearctic and India.

***Cheilosia latifrons* (Zetterstedt, 1843)**

**Literature records.** Rif (Becker 1894: 395, as *Chilosia marokkana*, Becker and Stein 1913: 87, as *Chilosia maroccana*, Gil Collado 1929: 405, Kassebeer 1998c: 60). Middle Atlas, High Atlas (Kassebeer 1998c). Cental Plateau (Timon-David 1951: 144, as *Chilosia intonsa*). Cited (Séguy 1961: 43, as *Chilosia maroccana*). Listed (Claussen 1989b: 372, as *Cheilosia maroccana*). Distribution map (Dirickx 1994: 34, 36, 159).

**New site.** Rif: Oued Sidi Yahya Aârab, 25/IV/2015, 1♂, sweep net, leg. Sahib and Belqat.

**Distribution.** Palaearctic.

***Cheilosia paralobi* Malski, 1962**

**Literature records.** Rif (Gil Collado 1929: 405, as *Cheilosia longula*). Middle Atlas (Claussen 1989b, Claussen and Hauser 1990: 436, Kassebeer 1998c: 61). High Atlas (Kassebeer 1998c: 61). Listed (Claussen 1989b: 372). Cited (Speight 2018: 44). Distribution map (Dirickx 1994: 38, 163).

**Distribution.** Portugal, Spain, Algeria and Morocco.

***Cheilosia parva* Kassebeer, 1998**

**Literature record.** Middle Atlas (Kassebeer 1998c: 62).

**Comment.** Endemic to Morocco.

**Distribution.** Morocco.

***Cheilosia rodgersi* Wainwright, 1911**

**Literature records.** Rif (Becker and Stein 1913: 88, Claussen 1989a: 283, Claussen 1989b: 372). Listed (Claussen 1989b: 372). Cited (Speight 2018: 48). Distribution map (Dirickx 1994: 40, 166).

**Distribution.** Portugal, southern Spain, and Morocco.

***Cheilosia ruffipes* (Zetterstedt, 1843)**

**Literature records.** Middle Atlas (Claussen and Hauser 1990: 436 as *Cheilosia soror*, Kassebeer 1998c: 65, as *Cheilosia rufipes*). Distribution map (Dirickx 1994: 42, 169).

**Distribution.** Palaearctic.

***Cheilosia scutellata* (Fallén, 1817)**

**Literature records.** Rif (Gil Collado 1929: 405, Kassebeer 1998c: 65). Middle Atlas (Kassebeer 1998c: 65). Listed (Claussen 1989b: 372). Distribution map (Dirickx 1994: 42, 169).

**Distribution.** Palaearctic.

***Cheilosia variabilis* (Panzer, 1798)**

**Literature record.** Middle Atlas (Kassebeer 1998c: 65). Cited (Speight 2018: 52).

**Distribution.** Palaearctic.

##### Genus *FERDINANDEA* Rondani, 1844

***Ferdinandea fumipennis* Kassebeer, 1999**

**Literature records.** Middle Atlas (Kassebeer 1999c: 153–162). High Atlas (Kassebeer 1999c). Cited (Speight 2018: 110).

**Distribution.** Portugal, Spain, France, Tunisia, and Morocco.

#### Tribe CHRYSOGASTERINI Shannon, 1922

##### Genus *BRACHYOPA* Meigen, 1822

***Brachyopa atlantea* Kassebeer, 2000**

**Literature record.** High Atlas (Kassebeer 2000: 141–148). Cited (Speight 2018: 9).

**Distribution.** Southern Spain and Morocco.

##### Genus *CHRYSOGASTER* Meigen, 1803

***Chrysogaster basalis* Loew, 1857**

**Literature records.** Middle Atlas (Claussen and Hauser 1990: 436, Kassebeer 1999b: 156). Distribution map (Dirickx 1994: 45, 173).

**New site.** High Atlas: Lac Oukaïmeden, 18/VI/2014, 1♂, sweep net, leg. Bot.

**Comment.** New record for the High Atlas.

**Distribution.** Western and Central Palaearctic.

##### Genus *IGHBOULOMYIA* Kassebeer, 1999

***Ighboulomyia atlasi* Kassebeer, 1999**

**Literature record.** Middle Atlas (Kassebeer 1999a: 11–24).

**New site.** Middle Atlas: Oued d’Ifrane, 10/VI/2014, 8♂♂, 3♀♀, sweep net, leg. Bot.

**Comments.** Monotypic genus *Ighboulomyia* with *Ighboulomyia atlasi*, described by Kassebeer (1999a). The genus *Ighboulomyia* was placed in the tribe Brachyopini. The genus is endemic to Morocco.

**Distribution.** Morocco.

##### Genus *MYOLEPTA* Newman, 1838

***Myolepta difformis* Strobl, 1909**

**Literature records.** High Atlas (Dirickx 1994: 93, as *Myolepta philonis*, Reemer et al. 2004: 558, 567). Cited (Speight 2018: 162).

**Distribution.** Portugal, Spain, Algeria, Tunisia, and Morocco.

##### Genus *NEOASCIA* Williston, 1886

***Neoascia clausseni*Hauser and Kassebeer 1998**

**Literature records.** Rif (Gil Collado 1929: 40, as *Neoascia podagrica*). Central Plateau (Părvu et al. 2006: 275). High Atlas (Hauser and Kassebeer 1998: 38–42). Listed (Claussen 1989b: 373, as *Neoascia podagrica*). Distribution map (Dirickx 1994: 94, 244).

**New sites.** Rif: Oued Jnane Niche, 14/VI/2013, 1♀; Oued Majjou (Hafa meqlouba), 10/V/2014, 1♀, sweep net, leg. Sahib and Belqat.

**Distribution.** Algeria, Tunisia, and Morocco.

##### Genus *ORTHONEVRA* Macquart, 1829

***Orthonevra bouazzai* Kassebeer, 1999**

**Literature record.** Middle Atlas (Kassebeer 1999a: 158–161).

**Comment**. Endemic to Morocco.

**Distribution.** Morocco.

**Figure 2.**
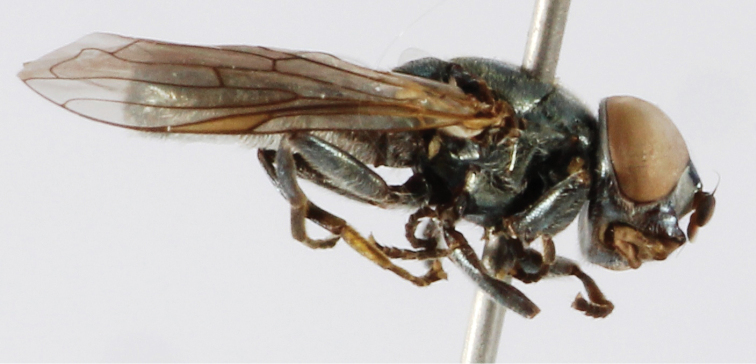
*Orthonevra brevicornis*, female, lateral view.

***Orthonevra brevicornis* (Loew, 1843) (Fig. 2)**

**Figure 3.**
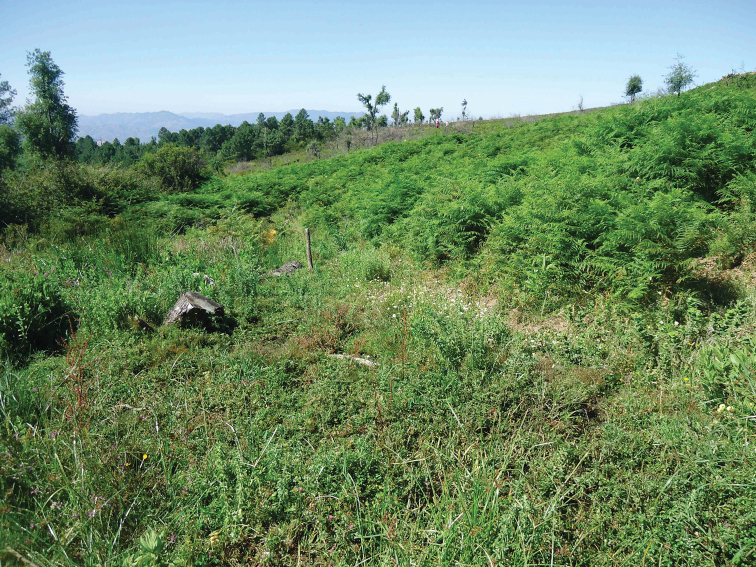
Habitat of *Orthonevra brevicornis*: Aïn Afersiw environment.

**New record**. Rif: Aïn Afersiw (Fig. 3), 11/VI/2013, 1♀, sweep net, leg. Sahib and Belqat.

**Comment.** New record for Morocco. In North Africa, the species was known only from Algeria (Djellab et al. 2013: 6). It prefers forests and wet woodland, standing-water bodies and flushes among *Quercus* forest, humid *Pinus sylvestris* forest and wet *Salix* woodland. It visits flowers such as Apiaceae, *Cornus*, *Crateagus*, *Malus*, *Pyrus communis*, *Ranunculus*, *Rorippa*, *Salix* (Speight 2018) in sunlit glades among woodland. The species is collected in the present study in a pond surrounded by Conifer reforestation, with the edges overgrown by grasses and herbaceous vegetation including fern, *Rubus* and *Cistus monspeliensis*.

**Distribution.** Western and Central Palaearctic.

***Orthonevra elegans* (Meigen, 1822)**

**Literature records.** Rif (Becker and Stein 1913: 87). Listed (Claussen 1989b: 373). Distribution map (Dirickx 1994: 96, 246).

**Distribution.** Palaearctic.

***Orthonevra schachti* Claussen, 1989**

**Literature records.** High Atlas (Claussen 1989b, Dirickx 1994: 97, Kassebeer 1999a: 160).

**Comments.** Endemic to Morocco.

**Distribution.** Morocco.

##### Genus *RIPONNENSIA* Maibach, Goeldin and Speight 1994

***Riponnensia longicornis* (Loew, 1843)**

**Literature records.** Central Plateau (Séguy 1961: 23, as *Orthonevra longicornis*). High Atlas (Kanervo 1939: 2, as *Orthonevra longicornis*, Kassebeer 1999a: 162). Middle Atlas (Kassebeer 1999a: 162). Cited (Speight 2018: 224). Listed (Claussen 1989b: 373). Distribution map (Dirickx 1994: 97, 247).

**Distribution.** Mediterranean Basin.

***Riponnensia splendens* (Meigen, 1822)**

**Literature records.** Rif (Gil Collado 1929: 405, as *Chrysogaster splendens*). Middle Atlas (Kassebeer 1999a). High Atlas (Claussen 1989b, as *Orthonevra splendens*, Kassebeer 1999a: 162). Recorded and listed (Claussen 1989b: 363, 373). Distribution map (Dirickx 1994: 97, 248 (as *Orthonevra splendens*)).

**New site.** Middle Atlas: Douar Zaouiat Cheikh, 19/III/2008, 2♂♂, sweep net, leg. A. van Eck.

**Distribution.** Western Palaearctic.

#### Tribe ERISTALINI Newman, 1834

##### Genus *ANASIMYIA* Schiner, 1864

***Anasimyia contracta* Claussen and Torp, 1980**

**Literature record.** Middle Atlas (Kassebeer 1998a: 22).

**Distribution.** Western Palaearctic.

##### Genus *ERISTALINUS* Rondani, 1845

***Eristalinus aeneus* (Scopoli, 1763)**

**Literature records.** Rif (Becker and Stein 1913: 86, as *Lumpetia aenea*, Gil Collado 1929: 406, 407 (as *Eristalis aeneus*), Leclercq 1961: 242, as *Eristalis aeneus*. Central Plateau (Timon-David 1951: 146, as *Lathyrophtalmus aeneus*, Gil Collado 1929, Séguy 1930: 129, as *Lathyrophtalmus aeneus*. High Atlas (Kanervo 1939: 5, as *Lathyrophtalmus aeneus*, Gill Collado 1929). Listed (Claussen 1989b: 372). Distribution map (Dirickx 1994: 53, 54, 190).

**New sites.** Rif: Jumb Kitane, 05/IV/2015, 1♂, sweep net, leg. Sahib and Belqat. High Atlas: Vicinity of Asni, 05/VI/2014, 1♀, sweep net, leg. Bot.

**Distribution.** Holarctic, Afrotropical, Oriental, and Australasian regions.

***Eristalinus megacephalus* (Rossi, 1794)**

**Literature records.** Rif (Becker and Stein 1913: 85, as *Eristalis quinquelineatus*, Gil Collado 1929: 407, as *Eristalis quinquelineatus*). Central Plateau (Séguy 1930: 129, as *Lathyrophthalmus quinquelineatus*). Cited (Séguy 1961: 200). Listed (Claussen 1989b: 372). Distribution map (Dirickx 1994: 54, 190).

**Distribution.** Western Palaearctic and Afrotropical regions.

***Eristalinus sepulchralis* (Linnaeus, 1758)**

**Literature records.** Rif (Becker and Stein 1913: 85, as *Eristalis sepulchralis*, Gil Collado 1929: 406, as *Eristalis sepulchralis*). Central Plateau (Gil Collado 1929). High Atlas (Claussen 1989b: 366, 372). Distribution map (Dirickx 1994: 54, 191).

**Distribution.** Palaearctic and India.

***Eristalinus taeniops* (Wiedemann, 1818)**

**Literature records.** Rif (Becker and Stein 1913: 85, as *Eristalis taeniops*). Central Plateau, High Atlas (Séguy 1930: 130). Middle Atlas (Leclercq 1961: 243). Listed (Claussen 1989b: 372). Cited (Speight 2018: 79). Distribution map (Dirickx 1994: 55, 191).

**New sites.** Rif: Oued Martil, 13/XII/2013, 1♀; Halouma Kitane, 1/I/2015, 1♂, sweep net, leg. Sahib and Belqat; Oued Sidi Yahya Aârab, 3/II/2015, 2♂♂, 1♀. Middle Atlas: Bridge Oued Oum Er-rabie (Douar Ahl Souss), 17/V/2017, 1♂, reared by Sahib and Belqat.

**Distribution.** Palaearctic, Oriental, and Afrotropical regions.

##### Genus *ERISTALIS* Latreille, 1804

***Eristalis arbustorum* (Linnaeus, 1758)**

**Literature records.** Rif (Becker and Stein 1913: 85, Gil Collado 1929: 407, Kanervo 1939: 4). Central Plateau (Timon-David 1951: 146). Middle Atlas (Claussen 1989b: 366, Timon-David 1951: 146, Séguy 1930: 129). Listed (Claussen 1989b: 372). Distribution map (Dirickx 1994: 55, 192).

**New sites.** Rif: Aïn Sidi Brahim Ben Arrif, 25/IV/2014, 1♂, sweep net, leg. Sahib and Belqat. Middle Atlas: Vicinity of Ifrane, 7/VI/2014, 1♀, sweep net, leg. Bot. High Atlas: Vicinity of Asni, 05/VI/2014, 2♂♂, 07/VI/2014, 1♀; Lac Oukaïmeden, 18/VI/2014, 3♂♂, 19/VI/2014, 1♂, 1♀, sweep net, leg. Bot. Anti Atlas: Douar Issafen, 12/IV/2015, 1♂; Douar Issafen, 14/IV/2015, 2♀♀, sweep net, leg. Schmid-Egger.

**Comment.** New records for the High Atlas and the Anti Atlas.

**Distribution.** Holarctic and Oriental regions.

***Eristalis pertinax* (Scopoli, 1763)**

**Literature records.** Central Plateau, Middle Atlas, High Atlas (Séguy 1930: 130). Listed (Claussen 1989b: 372). Distribution map (Dirickx 1994: 56, 194).

**Distribution.** Western Palaearctic.

***Eristalis similis* (Fallén, 1817)**

**Literature records.** Rif (Gil Collado 1929: 407, as *Eristalis pratorum*). High Atlas (Claussen 1989b: 366, 372). Cited (Séguy 1961: as *Eristalis pratorum*). Distribution map (Dirickx 1994: 56, 195).

**New sites.** Rif: Forest house, 18/V/2014, 1♂, 2♀♀, sweep net, leg. Sahib and Belqat. High Atlas: Douar Zaouiat Cheikh, 19/III/2008, 1♂, sweep net, leg. AvEck; Lac Oukaïmeden, 19/VI/2014, 1♀, sweep net, leg. Bot.

**Distribution.** Palaearctic.

***Eristalis tenax* (Linnaeus, 1758)**

**Literature records.** Rif (Becker and Stein 1913: 85, as *Erystalomyia tenax*, Gil Collado 1929: 407, Kanervo 1939: 5, Leclercq 1961: 242). Central Plateau (Gil Collado 1929: 407, Timon-David 1951, as *Erystalomyia tenax*). Middle Atlas (Kanervo 1939, Timon-David 1951: 146, Leclercq 1961). High Atlas (Gil Collado 1929, Kanervo 1939, Timon-David 1951). Anti Atlas (Séguy 1949: 156, Timon-David 1951: 146). Cited (Séguy 1930: 130). Listed (Claussen 1989b: 372). Distribution map (Dirickx 1994: 57, 195).

**New sites.** Rif: Village Sebt Zinnat, 15/III/2008, 1♀; Belyounech, 9/V/2013, 1♂; Aïn Takhninjoute, 17/V/2014, 2♂♂; Forest house, 18/V/2014, 1♀; Jumb Kitane, 18/IV/2018, 1♀; Meadow Fahs Lmhar, 15/IV/2018, 2♂♂, sweep net, leg. Sahib and Belqat. High Atlas: Douar Zaouiat Cheikh, 19/III/2008, 1♂, sweep net, leg. AvEck; Vicinity of Asni, 05/VI/2014, 1♀, sweep net, leg. Bot; Lac Oukaïmeden, 18/VI/2014, 2♂♂, sweep net, leg. Bot.

**Distribution.** Cosmopolitan, except Antarctica.

##### Genus *HELOPHILUS* Meigen, 1822

***Helophilus trivittatus* (Fabricius, 1805)**

**Literature records.** Rif (Becker and Stein 1913: 86, Gil Collado 1929: 407). Listed (Claussen 1989b: 372). Distribution map (Dirickx 1994: 70, 209).

**Distribution.** Palaearctic.

##### Genus *MALLOTA* Meigen, 1822

***Mallota cimbiciformis* (Fallen, 1817)**

**Literature records.** Rif (Becker and Stein 1913: 85, as *Mallota eristaloides*). Listed (Claussen 1989b: 373). Distribution map (Dirickx 1994: 73, 213).

**Distribution.** Western and Central Palaearctic.

***Mallota dusmeti* Andreu, 1926**

**Literature records.** High Atlas (Kassebeer 1998a: 23).

**Distribution.** Portugal, central Spain, Tunisia, and Morocco.

**Genus *MELANOGASTER* Róndani, 1857**

***Melanogaster lindbergi* Kassebeer, 1999**

**Literature records.** Rif (Becker and Stein 1913: 87, as *Chrysogaster macquarti*). Middle Atlas (Kanervo 1939: 2, as *Chrysogaster viduata*, Kassebeer 1999a: 158). Cited (Séguy 1961: 27, 28 (as *Chrysogaster viduata*)). Listed (Claussen 1989b: 372, as *Chrysogaster lucida*). Distribution map (Dirickx 1994: 46, 175 (as *Chrysogaster viduata*)).

**Comment.** Endemic to Morocco.

**Distribution.** Morocco.

##### Genus *MYATHROPA* Rondani, 1845

***Myathropa florea* (Linnaeus, 1758)**

**Literature records.** Rif (Becker and Stein 1913: 85, Gil Collado 1929: 407). Middle Atlas (Séguy 1930, Claussen 1989b: 366). Listed (Claussen 1989b: 373). Distribution map (Dirickx 1994: 92, 240).

**New site.** Rif: Oued 15 km from Fifi, 6/V/2014, 1♀, sweep net, leg. Sahib and Belqat.

**Distribution.** Palaearctic.

##### Genus *PARHELOPHILUS* Girschner, 1897

***Parhelophilus versicolor* (Fabricius, 1794)**

**Literature records.** Central Plateau (Gil Collado 1929: 407, as *Helophilus versicolor*). Listed (Claussen 1989b: 373). Distribution map (Dirickx 1994: 104, 258).

**Distribution.** Palaearctic.

#### Tribe EUMERINI Smirnov, 1924

##### Genus *EUMERUS* Meigen, 1822

***Eumerus amoenus* loew, 1848**

**Literature records.** Cited (Séguy 1961: 207, Speight 2018: 87). Listed (Claussen 1989b: 372). Distribution map (Dirickx 1994: 57, 196).

**New sites.** High Atlas: Douar Aourir, 8/IV/2015, 2♂♂. Anti Atlas: Beach of Tamelallt, 9/IV/2015, 1♂, sweep net, leg. Schmid-Egger.

**Comment.** New records for the Anti Atlas.

**Distribution.** Palaearctic.

***Eumerus barbarus* (Coquebert, 1804)**

**Literature records.** Rif (Gil Collado 1929: 412, as *Eumerus australis*, Becker and Stein 1913: 86). Middle Atlas (Claussen 1989b: 363). High Atlas (Gil Collado 1929: 412). Cited (Séguy 1961: 208, Speight 2018: 88). Listed (Claussen 1989b: 372). Distribution map (Dirickx 1994: 58, 196).

**Distribution.** Mediterranean Basin.

***Eumerus basalis* Loew, 1848**

**Literature records.** Middle Atlas (Séguy 1930: 130, as *Eumerus angusticornis*). Listed (Claussen 1989b: 372). Distribution map (Dirickx 1994: 58, 197).

**Distribution.** Western Palaearctic.

***Eumerus caballeroi* Gil-Collado, 1929**

**Literature records.** Central Plateau (Gil Collado 1929: 412–414). Listed Claussen (1989b: 372). Cited (Speight 2018: 89). Distribution map (Dirickx 1994: 59, 197).

**Distribution.** Spain and Morocco.

***Eumerus hungaricus* Szilády, 1940**

**Literature record.** Cited (Speight 2018: 92).

**Distribution.** Mediterranean Basin.

***Eumerus lunatus* (Fabricus, 1794)**

**Literature records.** Rif (Becker and Stein 1913: 86, as *Eumerus lunalatus*, Leclercq 1961: 243). Listed (Claussen1989b: 372). Cited (Speight 2018: 94). Distribution map (Dirickx 1994: 62, 200).

**Distibution.** Afrotropical Region, Morocco. The Europe records require confirmation.

***Eumerus melotus* (Séguy, 1941)**

**Literature records.** High Atlas (Séguy 1941: 13, as *Lampetia melota*). Listed (Claussen 1989b: 372). Distribution map (Dirickx 1994: 62).

**Comments**. Endemic to Morocco.

**Distribution.** Morocco.

***Eumerus nudus* Loew, 1848**

**Literature records.** Rif (Becker and Stein 1913: 86). Cited (Speight 2018: 95). Listed (Claussen 1989b: 372). Distribution map (Dirickx 1994: 64, 201).

**Distribution.** Mediterranean Basin, Romania, and former Yugoslavia.

**Figure 4.**
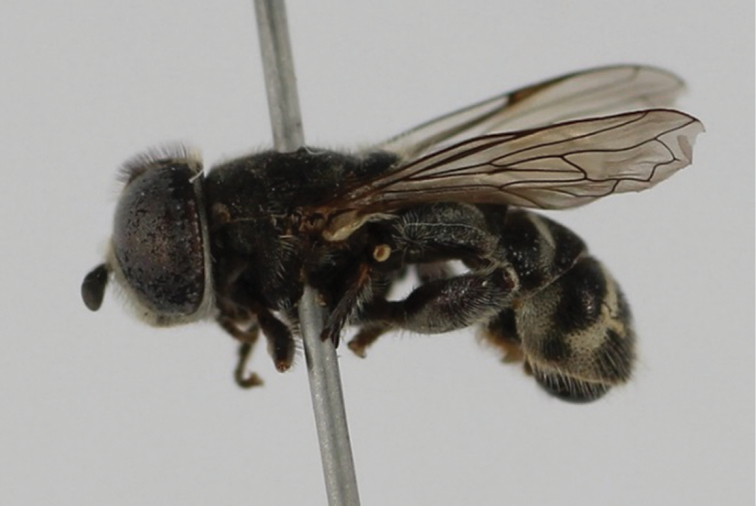
*Eumerus obliquus*, male, lateral view.

***Eumerus obliquus* (Fabricius, 1805) (Fig. 4)**

**Figure 5.**
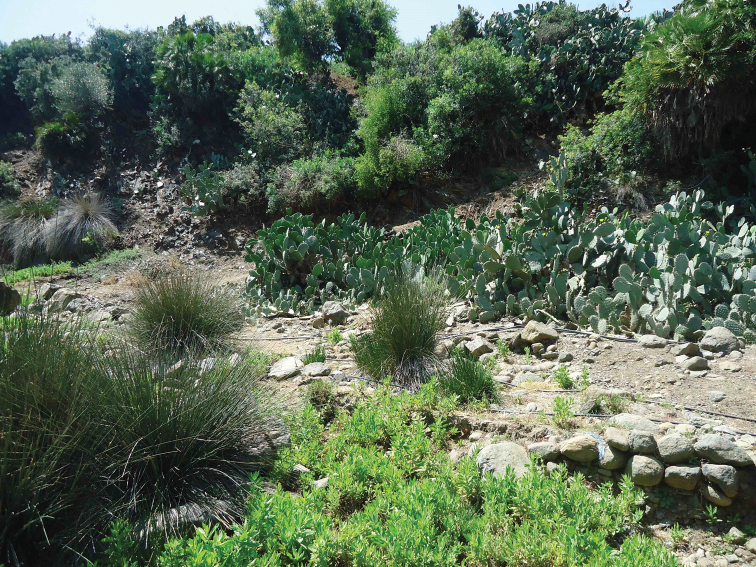
Habitat of *Eumerus obliquus*: Oued Jnane Niche environment.

**Figure 6.**
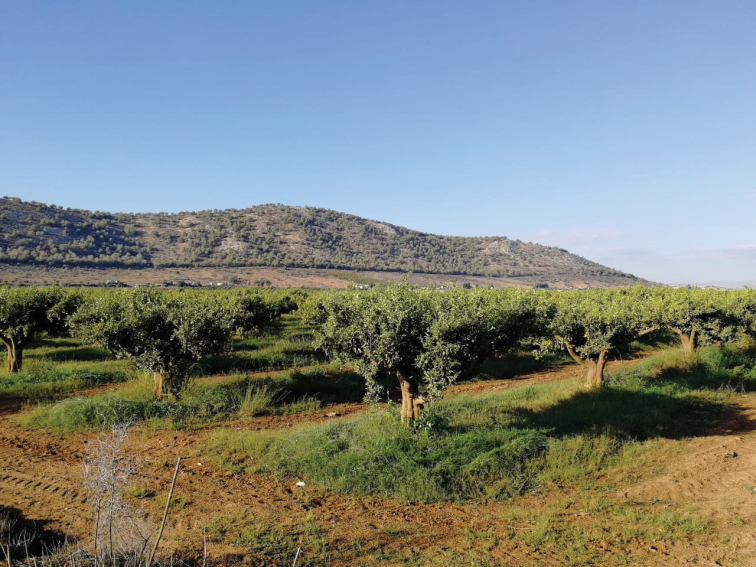
Habitat of *Eumerus obliquus*: Oued El Khemis environment.

**New records**. Rif: Oued Jnane Niche (Fig. 5), 25/IV/2015, 1♂, sweep net, leg. Sahib and Belqat. Eastern region: Oued El Khemis (Fig. 6), 11/IX/2013, 1♂, sweep net, leg. Sahib and Belqat.

**Comments.** New to Morocco. *E. obliquus* was recorded from Egypt (Steyskal and El-Bialy 1967, Peck 1988). The species is found in open ground, thinly vegetated, semi-arid dry grassland, often along the margins of seasonal rivers; it feeds from the juice of ripe, fallen fruits of *Opuntia* (Speight 2018). In Morocco, we collected adults by sweeping the vegetation around lotic habitats. As stated in the literature, we found the species in the margins of a temporary river. The wet section was reduced to a thin layer of water. Riparian vegetation consisted primarily of *Opuntia ficus indica* (Fig. 5), of *Nerium oleander*, and herbaceous vegetation. The second habitat was also a river flowing towards oued Moulouya with predominant vegetation consisted of *Olea europea*, *Citrus sinensis*, *Citrus reticulata*, *Punica granatum*, and *Eucalyptus*.

**Distribution.** Mediterranean Basin, the Canaries, Afrotropical, Malagasy, Australasian regions.

***Eumerus ornatus* Meigen, 1822**

**Literature records.** Middle Atlas (Séguy 1930: 130). Listed (Claussen 1989b: 372). Distribution map (Dirickx 1994: 64, 202).

**Distribution.** Western Palaearctic.

***Eumerus pulchellus* Loew, 1848**

**Literature records.** Cited (Séguy 1961: 211). Listed (Claussen 1989b: 372). Distribution map (Dirickx 1994: 65, 203).

**Distribution.** Western Palaearctic.

***Eumerus punctifrons* Loew, 1857**

**Literature records.** Rif (Leclercq 1961: 243). Listed (Claussen 1989: 372). Distribution map (Dirickx 1994: 65, 203).

**Distribution.** Afrotropical Region, southern part of the Mediterranean Basin.

***Eumerus pusillus* Loew, 1848**

**Literature records.** High Atlas (Claussen 1989b: 363, 372). Distribution map (Dirickx 1994: 66, 203).

**Distribution.** Mediterranean Basin.

***Eumerus sabulonum* (Fallén, 1817)**

**Literature records.** Cited (Séguy 1961: 212). Listed (Claussen 1989b: 372). Distribution map (Dirickx 1994: 66, 204).

**Distribution.** Western Palaearctic.

***Eumerus schmideggeri* Van Steenis, Hauser and Van Zuijen, 2017**

**Literature record.** Anti Atlas (Van Steenis et al. 2017: 155–158).

**Distribution.** Algeria, Tunisia, and Morocco.

***Eumerus strigatus* (Fallén, 1817)**

**Literature records.** Rif, High Atlas (Kanervo 1939: 5). Central Plateau (Timon-David 1951: 147). Listed (Claussen 1989b: 372). Distribution map (Dirickx 1994: 67, 205).

**Distribution.** Palaearctic, North America, Australia, and New Zealand.

***Eumerus subornatus* Claussen, 1989**

**Literature records.** High Atlas (Claussen 1989b: 364, 365, 372). Cited (Speight 2018: 100). Distribution map (Dirickx 1994: 68).

**Distribution.** Portugal, France, Spain, and Morocco.

***Eumerus truncatus* Rondani, 1868**

**Literature records.** Anti Atlas (Van Steenis et al. 2017: 160). Cited (Speight 2018: 102).

**Distribution.** Portugal, Spain, Greece, Italy, Tunisia, and Morocco.

##### Genus *PLATYNOCHAETUS* Wiedemann, 1830

***Platynochaetus rufus* Macquart, 1835**

**Literature records.** Central Plateau (Gil Collado 1929: 407). Distribution map (Dirickx 1994: 113, 271).

**Distribution.** Maltese islands, Algeria, and Morocco.

***Platynochaetus setosus* (fabricius, 1794)**

**Literature records.** High Atlas (Gil Collado 1929: 407). Listed (Claussen 1989b: 373). Cited (Speight 2018: 217). Distribution map (Dirickx 1994: 114, 272).

**Distribution.** Western part of the Mediterranean Basin.

#### Tribe MERODONTINI (Edwards, 1915)

##### Genus *MERODON* Meigen, 1803

***Merodon aberrans* Egger, 1860**

**Literature records.** Cited (Séguy 1961: 174, as *Lampetia aberrans*, Speight 2018: 132). Listed (Claussen 1989b: 373). Distribution map (Dirickx 1994: 77, 220).

**Distribution.** Western Palaearctic.

***Merodon arrasus* Beck, 1921**

**Literature records.** Rif (Becker 1921: 54, 55). Listed (Claussen 1989b: 373). Distribution map (Dirickx 1994: 78, 222).

**Distribution.** Tunisia, Algeria, and Morocco.

***Merodon bequaerti* Hurkmans, 1993**

**Literature records.** Middle Atlas; Eastern region (Vujić et al. 2020: 98).

**Comments.***Merodon bequaerti* belongs to the *Merodon serrulatus* species group (Vujić et al. 2020: 81); this species could be *Merodon alexeji* (Hurkmans 1993: 163).

**Distribution.** Algeria, Tunisia, Libya, and Morocco.

***Merodon biarcuatus* Curran, 1939**

**Literature records.** Central Plateau (Curran 1939: 6, 7). Listed (Claussen 1989b: 373). Cited (Speight 2018: 137). Distribution map (Dirickx 1994: 79).

**Distribution.** France, Italy, Greece, Turkey, and Morocco.

***Merodon cabanerensis* Marcos-García, Vujić and Mengual, 2007**

**Literature records.** High Atlas (Vujić et al. 2018: 113, 114, 135). Cited (Speight 2018: 137).

**Distribution.** Spain and Morocco.

***Merodon chalybeus* Wiedemann in Meigen, 1822**

**Literature records.** Central plateau (Timon-David 1951: 146, as *Lampetia spicata*). Cited (Claussen 1989b: 365, 373 (as *Merodon spicatus*), Marcos-García et al. 2007: 546, Speight 2018: 138). Cited (Speight 2018: 138). Distribution map (Dirickx 1994: 87, 233).

**Distribution.** Mediterranean Basin.

***Merodon clavipes* (Fabricius, 1781)**

**Literature records.** Cited (Hurkmans 1993: 178, 230, Marcos-García et al. 2007: 548, Speight 2018: 138).

**Distribution.** Western Palaearctic.

***Merodon constans* (Rossi, 1794)**

**Literature records.** Rif (Becker and Stein 1913: 86, as *Lampetia constans*). Listed (Claussen 1989b: 373). Distribution map (Dirickx 1994: 81, 226).

**Distribution.** Western Palaearctic.

***Merodon distinctus* Palma, 1863**

**Literature records.** Middle Atlas, Central Plateau (Timon-David 1951: 146, as *Lampetia distincta*). Listed (Claussen 1989b: 373). Distribution map (Dirickx 1994: 81, 226).

**Distribution.** Mediterranean Basin.

***Merodon elegans* Hurkmans, 1993**

**Literature records.** Central Plateau (Hurkmans 1993: 195). Cited (Speight 2018: 141).

**Distribution.** Western part of Mediterranean Basin.

***Merodon eques* Fabricius, 1805**

**Literature records.** Cited (Séguy 1961: 178, as *lampetia eques*). Listed (Claussen 1989b: 373). Distribution map (Dirickx 1994: 81, 227).

**Distribution.** Mediterranean Basin.

***Merodon equestris* (Fabricius, 1794)**

**Literature records.** Central Plateau (Fabricius 1805: 240, as *Eristalis ferrugineus*). Cited (Séguy 1961: 178, 179 (as *Lampetia equestris*)). Listed (Claussen 1989: 373). Distribution map (Dirickx 1994: 81, 227).

**Distribution.** Holarctic.

***Merodon escalerai* Gil Collado, 1929**

**Literature records.** Central Plateau (Gil Collado 1929: 408, 409). Listed (Claussen 1989b: 373). (Dirickx 1994: 82).

**Distribution.** Spain and Morocco.

***Merodon geniculatus* Strobl, 1909**

**Literature records.** Rif (Gil Collado 1929: 408). Listed (Claussen 1989b: 365, 373). Cited (Speight 2018: 144). Distribution map (Dirickx 1994: 83, 229).

**New Site.** High Atlas: Lac Oukaïmeden, 18/VI/2014, 2♂♂, 1♀, 19/VI/2014, 6♂♂, sweep net, leg. Bot.

**Comment.** New record for the High Atlas.

**Distribution.** Mediterranean Basin.

***Merodon ibericus* Vujíc, 2015**

**Literature records.** Rif (Becker and Stein 1913: 86, as *Lampetia spinipes*, Gil Collado 1929: 409, as Merodon spinipes var. avidus, Kanervo 1939: 5). Central Plateau (Séguy 1930: 130, Timon-David 1951: 146, as *Lampetia spinipes*). Middle Atlas (Séguy 1930: 130, Kanervo 1939: 5, as Lampetia spinipes var. avida, Popović et al. 2015: 796, Acanski et al. 2016: 3). High Atlas (Gil Collado 1929, Claussen 1989b). Cited (Séguy 1961: 176, as *Lampetia avida*, Speight 2018: 145). Listed (Claussen 1989: 365, 373). Distribution map (Dirickx 1994: 79, 223).

**New site.** High Atlas: Lac Oukaïmeden, 18/VI/2014, 1♂, 1♀, 19/VI/2014, 1♂, sweep net, leg. Bot.

**Distribution.** Portugal, Spain, and Morocco.

***Merodon italicus* Rondani, 1845**

**Literature records.** Middle Atlas (Claussen and Hauser 1990: 437, as *Merodon longicornis*). Distribution map (Dirickx 1994: 84, 230).

**Distribution**. Mediterranean Europe, southern Russia, Lebanon, and Morocco.

***Merodon maroccanus* Gil Collado, 1929**

**Literature records.** Central Plateau (Gil Collado 1929: 410, 411). Listed (Claussen 1989b: 373). Distribution map (Dirickx 1994: 84).

**Comments.** Endemic to Morocco.

**Distribution.** Morocco.

***Merodon minutus* Strobl, 1893**

**Literature records.** Middle Atlas (Séguy 1961: 180, as *lampetia minutus*). Listed (Claussen 1989b: 373). Cited (Speight 2018: 149). Distribution map (Dirickx 1994: 84, 230).

**Distribution.** Southern France around the Mediterranean coast to the Balkan Peninsula.

***Merodon murorum* (Fabricius, 1794)**

**Literature records.** Central Plateau (Fabricius 1794: 288, as *Syrphus murorum*, Meigen 1830: 354, as *Merodon auripilus*). High Atlas (Séguy 1941: 13, as *Lampetia auripila*). Cited (Séguy 1961: 176). Listed (Claussen 1989b: 373). Distribution map (Dirickx 1994: 79, 222).

**Comments.** Revised by Vujić et al. (2018: 123–125). *Merodon auripilus* is a junior synonym of *M. murorum*.

**Distribution.** Algeria, Tunisia, and Morocco.

***Merodon pruni* (Rossi, 1790)**

**Literature records.** Rif (Becker and Stein 1913: 86, as *Lampetia pruni*, Gil Collado 1929: 407, 408 (as Merodon pruni var. obscurus)). Listed (Claussen 1989b: 373). Distribution map (Dirickx 1994: 85, 231).

**Distribution.** Western Palaearctic, including Turkmenistan and Iraq.

***Merodon pumilus* Macquart, 1849**

**Literature records.** Rif (Becker and Stein 1913: 86, as *Lampetia aenea*, Kanervo 1939: 5, as *Lampetia aenea*, Ebejer et al. 2019: 150). Central Plateau (Gil Collado 1929: 407). Middle Atlas (Séguy 1930: 130). Cited (Séguy 1961: 174). Listed (Claussen 1989b: 373). Distribution map (Dirickx 1994: 77, 220).

**Distribution**. Spain, Algeria, and Morocco.

***Merodon rufus* Meigen, 1838**

**Literature records.** Rif (Becker and Stein 1913: 86, as *Lampetia rufa*). Listed (Claussen 1989b: 373). Distribution (Dirickx 1994: 86, 232).

**Distribution.** Western and Central Palaearctic.

***Merodon segetum* (Fabricius, 1794)**

**Literature records.** Cited (Peck 1988: 173). Listed (Claussen 1989b: 373). Distribution map (Dirickx 1994: 86, 232).

**Distribution.** Spain, Gibraltar, Macedonia, Crete, Algeria, and Morocco.

***Merodon sophron* Hurkmans, 1993**

**Literature records.** Middle Atlas (Hurkmans 1993: 168, Vujic et al. 2020: 135–137).

**Comment.** Endemic to Morocco. *Merodon sophron* is one of the 13 species belonging to the *Merodon serrulatus* species group. It is redefined and redescribed by Vujic et al. (2020) on the material from the type locality of Azrou.

**Distribution.** Morocco.

***Merodon tangerensis* Hurkmans, 1993**

**Literature record.** Rif (Hurkmans 1993: 172).

**Comment.** Endemic to Morocco.

**Distribution.** Morocco.

***Merodon tricinctus* Sack, 1913**

**Literature records.** High Atlas, Anti Atlas (Kassebeer 1998a: 24). Middle Atlas (Timon-David 1951: 146, as *Lampetia tricincta*). Listed (Claussen 1989b: 373). Distribution map (Dirickx 1994: 88, 234).

**Distribution.** Western Palaearctic.

**Figure 7.**
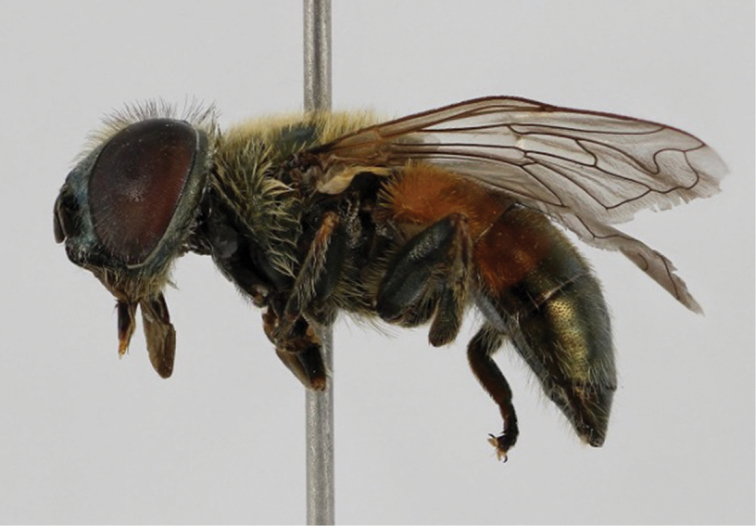
*Merodon unguicornis*, female, lateral view.

***Merodon unguicornis* Strobl 1909 (Fig. 7)**

**Literature record.** Middle Atlas (Ebejer et al. 2019: 150).

**New site**. Rif: Forest house, 17/V/2014, 1♀, sweep net, leg. Sahib and Belqat.

**Figure 8.**
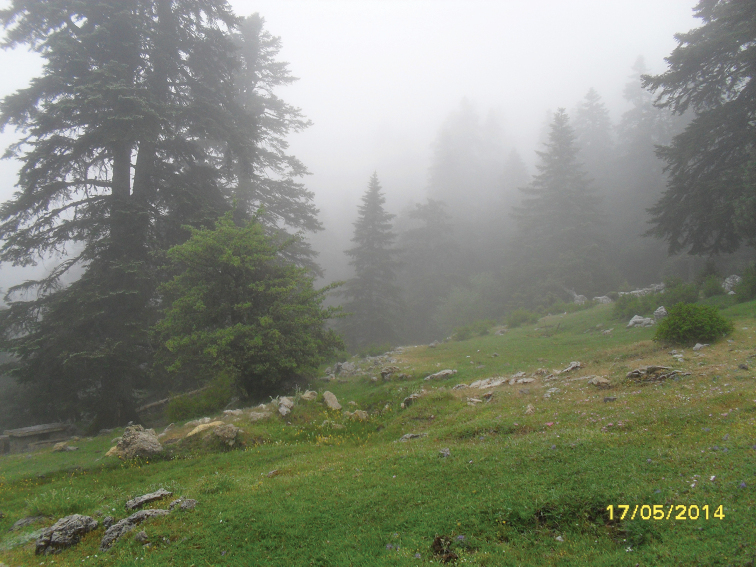
Habitat of *Merodon unguicornis*: Forest house environment.

**Comment.** New record for the Rif. *M. unguicornis* was cited for the first time in Morocco by Ebejer et al. (2019). In the present work, the female was collected at the locality of Forest house environment (Fig. 8), crossed by a spring and a brook and where the most predominant vegetation was formed by *Abies marocana*, *Pinus negra*, *Pinus pinaster*, *Cedrus atlantica* and *Berberis hispanica*. Collected on a misty day, as visualized in Figure 8.

**Distribution.** Spain and Morocco.

##### Genus *PSILOTA* Meigen, 1822

***Psilota atra* (Fallén, 1817)**

**Literature records.** High Atlas (Kassebeer 1995b: 395–400, as *Psilota toubkalana*). Cited (Smit and Vujić 2008: 350, Speight 2018: 220).

**New Site.** Middle Atlas: Douar Zaouiat Cheikh, 19/III/2008, 1♀, sweep net, leg. A. van Eck.

**Comment.** New record for the Middle Atlas.

**Distribution.** Europe and Morocco.

#### Tribe VOLUCELLINI Newman, 1834

##### Genus *VOLUCELLA* Geoffroy, 1762

***Volucella inanis* (Linnaeus, 1758)**

**Literature records.** Middle Atlas (Claussen and Hauser 1990: 436). Distribution map (Dirickx 1994: 126, 289).

**Distribution.** Palaearctic.

***Volucella liquida* Erichson in Wagner, 1841**

**Literature records.** Rif (Gil Collado 1929: 406, Leclercq 1961: 242). Central Plateau (Timon-David 1951: 146, Séguy 1930: 129). Middle Atlas (Kanervo 1939: 4, Timon-David 1951: 146, Séguy 1953: 84, Leclercq 1961). High Atlas (Séguy 1930: 129). Listed (Claussen 1989b: 374). Distribution (Dirickx 1994: 127, 289).

**New site.** Rif: Jumb Kitane, 19/IX/2017, 1♀, sweep net, leg. Sahib and Belqat.

**Distribution.** Algeria and Morocco.

***Volucella zonaria* (Poda, 1761)**

**Literature records.** Central Plateau (Séguy 1930: 129). Listed (Claussen 1989b: 374). Distribution map (Dirickx 1994: 127, 290).

**Distribution.** Palaearctic.

#### Tribe XYLOTINI Bigot, 1883

##### Genus *BRACHYPALPUS* Macquart, 1834

***Brachypalpus valgus* (Panzer, 1798)**

**Literature records.** High Atlas, Middle Atlas (Kassebeer 1998a: 22).

**Distribution.** Western Palaearctic.

##### Genus *MILESIA* Latreille, 1804

***Milesia crabroniformis* (Fabricius, 1775)**

**Literature records.** Middle Atlas (Claussen and Hauser 1990: 437). Distribution map (Dirickx 1994: 92, 240).

**Distribution.** Europe, Turkey, Georgia, and Morocco.

##### Genus *SPILOMYIA* Meigen, 1803

***Spilomyia maroccana* Kuznetsov, 1997**

**Literature records.** Rif (Becker and Stein 1913: 86, as *Spilomyia digitate*, Kuznetzov 1997: 203–205). High Atlas (Claussen 1989b: 366). Listed (Claussen 1989b: 373, as *Spilomyia digitata*). Distribution map (Dirickx 1994: 123, 283 (as *Spilomyia digitata*)).

**Distribution.** Algeria and Morocco.

##### Genus *SYRITTA* Lepeletier and Serville, 1828

***Syritta flaviventris* Macquart, 1842**

**Literature records.** Central Plateau (Claussen 1989b: 366). Listed (Claussen 1989b: 373). Distribution map (Dirickx 1994: 123, 284).

**New site.** Eastern region: Farm Saf-Saf, 14/VI/2013, 1♂, 1♀, sweep net, leg. Sahib and Belqat.

**Comment**. New record for the Eastern region.

**Distribution.** Mediterranean Basin, Nearctic, Neotropical, Afrotropical regions, Madagascar and the Mascarene islands.

***Syritta pipiens* (Linnaeus, 1758)**

**Literature records.** Rif (Becker and Stein 1913: 88, Gill Collado 1929: 412, Kanervo 1939: 5). Central Plateau (Séguy 1930: 130, Timon-David 1951, Pârvu et al. 2006: 275). Middle Atlas (Séguy 1930). High Atlas (Timon-David 1951: 146, Claussen 1989b: 366). Listed (Claussen 1989b: 373). Distribution map (Dirickx 994: 124, 285).

**New sites.** Rif: Bni Maaden, 5/XI/2013, 1♂; Dam Moulay Bouchta, 5/IV/2014, 1♀; Oued El Koub, 30/V/2014, 1♂, 1♀; Douar Kitane, 18/VI/2014, 2♀♀; Oued Majjou (Hafa meqlouba), 27/IV/2015, 1♂, sweep net, leg. Sahib and Belqat. Eastern region: Farm Saf-Saf, 14/VI/2013, 4♂♂, sweep net, leg. Sahib and Belqat. Middle Atlas: Vicinity of Ifrane, 09/VI/2014, 1♀, sweep net, leg. Bot. High Atlas: Vicinity of Asni, 07/VI/2014, 5♂♂; Tizi N’Test, 15/VI/2014, 1♂, sweep net, leg. Bot. Anti Atlas: Oued Assa, 21/V/2015, 1♂, sweep net, leg. Sahib and Belqat.

**Comment.** New records for the Eastern region and the Anti Atlas.

**Distribution.** Holarctic, South America, and Indomalayan Region.

##### Genus *TEMNOSTOMA* Lepeletier and Serville, 1828

***Temnostoma bombylans* (Fabricius, 1805)**

**Literature records.** Middle Atlas (Séguy 1961: 155). Listed (Claussen 1989b: 374). Distribution map (Dirickx 1994: 125, 286).

**Distribution.** Palaearctic.

##### Genus *XYLOTA* Meigen, 1822

***Xylota segnis* (Linnaeus, 1758)**

**Literature records.** Rif (Becker and Stein 1913: 86, as Zelima (Xylota) segnis, Gil Collado 1929: 412, Leclercq 1961: 243). High Atlas (Timon-David 1951: 147). Listed (Claussen 1989b: 372). Distribution map (Dirickx 1994: 130, 293).

**New site.** Rif: Aïn El Maounzil, 17/V/2014, 1♀; Oued El Koub, 30/V/2014, 1♀; Oued Sidi Ben Sâada, 6/V/2015, 1♂, sweep net, leg. Sahib and Belqat. High Atlas: Vicinity of Asni, 07/VI/2014, 1♂, sweep net, leg. Bot.

**Distribution.** Palaearctic and eastern North America.

### Subfamily *PIPIZINAE* Mengual, Santos and Rojo 2015

#### Tribe PIPIZINI Williston, 1885

##### Genus *HERINGIA* Rondani, 1856

***Heringia heringi* (Zetterstedt, 1843)**

**Literature records.** High Atlas, Middle Atlas (Kassebeer 1998a: 23).

**Distribution.** Palaearctic.

##### Genus *PIPIZELLA* Rondani, 1856

***Pipizella thapsiana* Kassebeer, 1995**

**Literature records.** High Atlas (Kassebeer 1995a: 261–263). Cited (Speight 2018: 199).

**New site.** Middle Atlas: Douar Zaouiat Cheikh, 19/III/2008, 1♂, sweep net, leg. A. van Eck.

**Comment.** New record for the Middle Atlas.

**Distribution.** Portugal and Morocco.

##### Genus *TRIGLYPHUS* Loew

***Triglyphus escalerai* Gil Collado, 1929**

**Literature records.** Rif (Gil Collado 1929: 404). Cited (Speight 2018: 253). Distribution map (Dirickx 1994: 125).

**Distribution.** Portugal, Croatia, Montenegro, and Morocco.

## Discussion

The present work provides an important contribution to the Moroccan hoverfly fauna with two species recorded for the first time for Morocco: *Eumerus obliquus* (Fabricius, 1805) and *Orthonevra brevicornis* (Loew, 1843); nine new records are provided for the Rif, five for the Middle Atlas, and seven species are recorded for the first time for the Hight Atlas, the Anti Atlas, and the Eastern Region for which we have enlarged the distribution area. The 150 Moroccan species belong to three subfamilies, 14 tribes, and 49 genera (30 genera of Eristalinae, 16 of Syrphinae, and 3 of Pipizinae).

Knowledge of the hoverfly diversity of Morocco is unequal among regions. For instance, the Middle Atlas and the High Atlas have the same number of species (69), the Rif has 68 species, the Anti Atlas has 18 species, while the Eastern region has only seven species.

Four very common Palaearctic species were collected abundantly: *Episyrphus balteatus* (156 specimens), *Sphaerophoria scripta* (146 specimens), *Eupeodes corollae* (86 specimens) and *Melanostoma milinum* (78 specimens).

Morocco is home to 12 endemic species: two are endemic to the Rif, four to the Middle Atlas (including both an endemic genus and species (*Ighbouloumia atlasi*)), three to the High Atlas, one to the central plateau, and two species, *Platycheirus atlasi* and *Melanogaster lindbergi*, to the Middle Atlas plus the High Atlas, and to the Rif plus the Middle Atlas, respectively.

Morocco with the greatest number of total species (150), has 75% of the North African syrphid fauna. Algeria, with 91 species, has 46% of the total fauna. Tunisia, with 69, has 35% of the fauna, whereas Egypt and Libya have 51 species (26%) and 34 species (17%), respectively.

Algeria, Tunisia, Libya, and Egypt share with Morocco 71, 55, 25, and 30 species, respectively, but these numbers are likely to change and a detailed comparison must await a systematic sampling of the whole region.

Of the two Iberian Peninsula countries, Spain has the best known Syrphiidae fauna with 417 species whereas Portugal has only 195 species (Marcos-Garcia et al. 2002, Speight 2015, van Eck 2011, 2016).

These new data on the Moroccan hoverfly fauna reflect the variety of suitable habitats and suggest that many more species can yet be found in Morocco, that provides a wide variety of geographical and climatic properties. New species are also likely to be discovered by genomic prospecting using molecular approaches.
